# Validation of energy valley optimization for adaptive fuzzy logic controller of DFIG-based wind turbines

**DOI:** 10.1038/s41598-024-82382-y

**Published:** 2025-01-03

**Authors:** Basem E. Elnaghi, Ahmed M. Ismaiel, Fathy El Sayed Abdel-Kader, M. N. Abelwhab, Reham H. Mohammed

**Affiliations:** 1 Electrical Power and Machines Department, Faculty of Engineering, Suez Canal University, Ismailia, 41522 Egypt; 2 Electrical Power and Machine Department, Faculty of Engineering, Menoufia University, Menoufia, 32611 Egypt; 3 Electrical Computer and Control Engineering Department, Faculty of Engineering, Suez Canal University, Ismailia, 41522 Egypt

**Keywords:** Energy valley optimizer algorithm, Chaotic billiards optimization approach, Adaptive fuzzy logic controller, Double Fed induction generator, Grid-tied wind power plant, And Maximum Power Point Tracking (MPPT), Electrical and electronic engineering, Energy grids and networks

## Abstract

This study presents a novel optimization algorithm known as the Energy Valley Optimizer Approach (EVOA) designed to effectively develop six optimal adaptive fuzzy logic controllers (AFLCs) comprising 30 parameters for a grid-tied doubly fed induction generator (DFIG) utilized in wind power plants (WPP). The primary objective of implementing EVOA-based AFLCs is to maximize power extraction from the DFIG in wind energy applications while simultaneously improving dynamic response and minimizing errors during operation. The performance of the EVOA-based AFLCs is thoroughly investigated and benchmarked against alternative optimization techniques, specifically chaotic billiards optimization (C-BO), genetic algorithms (GA), and marine predator algorithm (MPA)-based optimal proportional-integral (PI) controllers. This comparative analysis is crucial in establishing the efficacy of the proposed method. To validate the proposed approach, experimental assessments are conducted using the DSpace DS1104 control board, allowing for real-time application of the control strategies. The results indicate that the EVOA-AFLCs outperform the C-BO-based AFLCs, GA-based AFLCs, and MPA-based optimal PIs in several key performance metrics. Notably, the EVOA-AFLCs exhibit rapid temporal response, a high rate of convergence, reduced peak overshoot, diminished undershoot, and significantly lower steady-state error. The EVOA-AFLC outperforms the C-BO-AFLC and GA-AFLC in terms of efficiency, transient responses, and oscillations. In comparison to the MPA-PI, it improves speed tracking by 86.3%, the GA-AFLC by 56.36%, and the C-BO by 39.3%. Moreover, integral absolute error (IAE) for each controller has been calculated to validate the system wind turbine performance. The EVOA-AFLC outperforms other approaches significantly, achieving a 71.2% reduction in average integral absolute errors compared to the GA-AFLC, 24.4% compared to the C-BO-AFLC, and an impressive 84% compared to the MPA-PI. These findings underscore the potential of the EVOA as a robust and effective optimization tool for enhancing the performance of adaptive fuzzy logic controllers in DFIG-based wind power systems.

## Introduction

Oil remains a dominant energy source for transportation, but its use contributes significantly to emissions and environmental degradation. In recent years, the transportation sector has experienced a sharp increase in emissions, and global logistics activities are projected to keep expanding. Concerns about fossil fuel consumption include rising supply costs and climate change. Wind energy has emerged as a leading clean energy technology due to its efficiency, cost-effectiveness, and minimal environmental impact. Since the inception of grid-connected wind turbines, power electronic converters have played a crucial role, with advancements over time^1–3^.

**Fig. 1:**
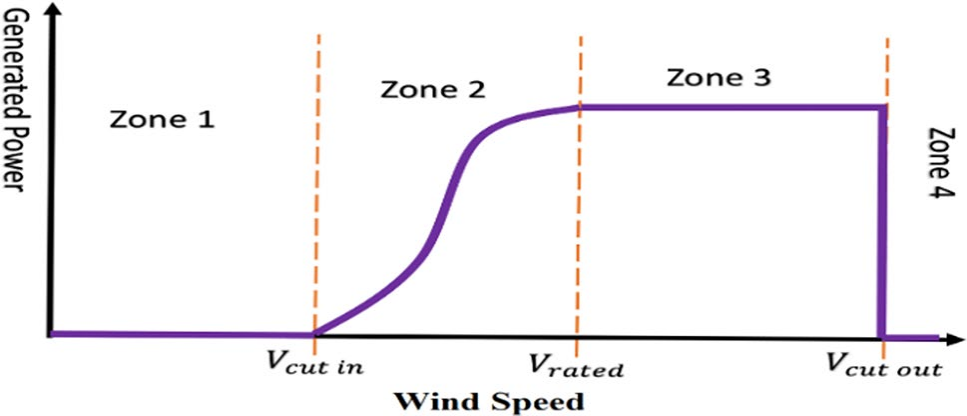
Operation of wind turbine.

Commercial wind turbines (WTs) employ various configurations of wind generators and power electronic converters (PECs) to achieve different operational modes: fully variable-speed (FVSWT), semi-variable-speed (SVSWT), and fixed-speed (FSWT). The FSWT, which relies on a PEC for initiation, is now considered outdated. Variable-speed wind turbines (VSWTs) offer enhanced power quality, efficiency, and compliance with grid codes, making them preferable over fixed-speed systems. VSWTs capture peak energy more effectively, resulting in higher power collection, reduced losses, and lower mechanical stress^4,5^. Figure 1 shows how a variable wind speed turbine operates^6^. Four zones can be distinguished within the operation. Zone 1 occurs when the wind speed is below the cut speed, making it impossible for the WPP to produce electricity. Zone 2 is referred to as the MPPT operation zone, where the pitch angle is maintained constant and the electromagnetic torque is managed to maximize WPP power. The pitch angle is controlled to maintain a steady power in zone 3, often known as the pitch control zone. When the wind speed reaches Zone 4, the turbine uses its emergency mechanism to shut down^7,8^, and^9^. This study focuses on Zone 2, in which MPPT is significant.

TSR control is an effective way to achieve MPPT for WPP. TSR is simpler and more productive under rapidly altering atmospheric conditions than other control systems^6–9^. TSR requires a speed sensor, which raises the price. Thus, wind speed estimation (WSE) is introduced in^6^. This method employs complex polynomial approximation equations, which reduces system accuracy^8,9^, and^10^. Therefore, TSR control is used during this research.

Currently, a range of electrical machine-based wind power plants (WPPs) is available^5,10–12^. Recent literature has explored various methods to improve the performance of grid-integrated WPPs, including pitch angle control (PAC), energy storage system (ESS) management, and voltage source converter (VSC) control^12–14^. While PAC is a mechanical controller with slower response times, ESS, despite its high cost and rated energy, has limitations. VSCs are favored for their quick and effective response without additional costs. The cascaded control scheme (CCS) is a strong contender for fine-tuning grid-tied converters^12,15^. Different controller types, such as proportional-integral (PI) regulators^10,16^ and fuzzy logic controllers (FLCs)^15,17^, have been successfully employed in CCS. This study reveals a notable research gap in the performance evaluation of Adaptive Fuzzy Logic Controllers (AFLCs) optimized using the Energy Valley Optimizer Approach (EVOA) compared to other contemporary techniques like chaotic billiards optimization (C-BO), genetic algorithms (GA), and marine predator algorithms (MPA). While PI controllers are prevalent in commercial applications due to their simplicity, robustness, and broad stability margins, they are inherently susceptible to instability caused by system nonlinearity and variability^13,18^. In contrast, AFLCs enhance control performance by integrating fuzzy logic to optimize PI controller inputs, effectively addressing these limitations^19–25^. AFLCs offer compelling advantages, such as their ability to manage system uncertainties, their model-free design, and their straightforward implementation. Despite these benefits, several methods have been proposed for designing fuzzy logic controllers in grid-integrated renewable energy control strategies, including Improved Arithmetic Optimization Algorithm (IAOA)^26,27^, the slap optimizer algorithm (SOA)^22^, whale optimizer algorithm (WOA)^28^, adaptive p-norm algorithm^29^, Antlion optimizer algorithm (AOA)^30^, and adaptive neuro-FLC combined with genetic algorithms (GAs)^31,32^. In recent years, diverse optimization techniques have been applied to fine-tune AFLCs. These include genetic algorithms (GA)^33–35^ grey wolf optimization^36^, whale optimizer^37^, and various meta-heuristic algorithms such as cuckoo search^38^, particle swarm optimization^39,40^, and the bees algorithm^35^. A significant advancement came with the introduction of the billiards optimizer algorithm (BOA) by Kaveh et al. in 2020^36^, which was subsequently improved by integrating chaotic logistic mappings (CLMs) to form the chaotic billiards optimization (C-BO) technique^37^. This integration enhances the algorithm’s performance by optimizing initialization, resulting in faster convergence and reduced computational demands.

Authors in^21^ present the use of the proportional integral optimized using marine predators algorithm (MPA-PI), a nature-inspired metaheuristic optimization technique, to enhance system response and power extraction. MPI-PI falls in slow convergence rate and large computational burden. Authors in^19^ investigated the AFLC to adjust MPPT only without adjusting DC link voltage or reactive power control using Matlab/Simulink and Dspace 1104. Authors in^44^ investigated the optimization of AFLC using different algorithms. AFLC-C-BO suffers from sensitivity to initial conditions, and complex tuning requirements. The Energy Valley Optimizer Algorithm (EVOA), introduced in January 2023 by Hadi and colleagues, is a promising new meta-heuristic approach^45^. EVOA-AFLC excels in achieving competitive outcomes with rapid convergence and minimal objective function evaluations compared to other algorithms such as GA-AFLC, C-BO-AFLC, and MPA-PI. This research gap emphasizes the need to explore the comparative performance of the EVOA in optimizing AFLCs against these established techniques. Investigating how EVOA stacks up against methods like C-BO-AFLC, GA-AFLC, and MPA-PI in practical applications can provide deeper insights into its efficacy and potential advantages. Such an exploration could lead to more robust and efficient control strategies for grid-integrated WPPs, addressing the limitations of current optimization approaches. This study employs the widely used tip speed ratio (TSR) control method for maximum power point tracking (MPPT) due to its accuracy and efficiency under varying atmospheric conditions. EVOA and C-BO are used to adjust the gains of adaptive fuzzy logic controllers (AFLCs) in the converters’ electronic switches, optimizing the grid-integrated WPPs. This paper introduces novel C-BO and EVOA methods to enhance the cascaded AFLC technique for grid-tied WPPs, demonstrating rapid convergence and improved performance in simulations. To our best of our knowledge in renewable energy literature or the literature on power system has discussed the EVOA-based optimal AFLC. The essential contributions to this article are illustrated as follows:

1. The main purpose of rotor-side converter (RSC) is to establish the MPPT by forcing the DFIG to operate at the optimum tip speed ratio. And, the grid-side converter (GSC) is used to adjust the DC link voltage, and power factor. The DC link voltage between GSC and RSC is adjusted at constant value for the most efficient operation of DFIG, and power factor is adjusted to unity by controlling the reactive power to zero. So, the implementation of AFLCs optimized using Energy Valley Optimizer Approach (EVOA) for both GSC and RSC is done to achieve maximum power point tracking, reactive power control, and DC link voltage control for DFIG used in WPP with minimal errors.
2. To show the viability of the EVOA-AFLC methodology against C-BO-based AFLCs, MPA-PI, and GA-AFLCs, and PIs techniques, a wind turbine performance index based on the gross system integral absolute error (IAE) is provided under identical wind turbine conditions.
3. Evaluation of the system using Matlab/Simulink 2022a, demonstrating the enhanced performance and robustness of EVOA-AFLCs compared to C-BO-based AFLCs, MPA-PI, and GA-AFLCs.
4. Experimental validation using DSpace DS1104, confirming the improvements and robustness of EVOA-AFLCs over C-BO-based AFLCs and GA-AFLCs.

## Model setup

**Fig. 2:**
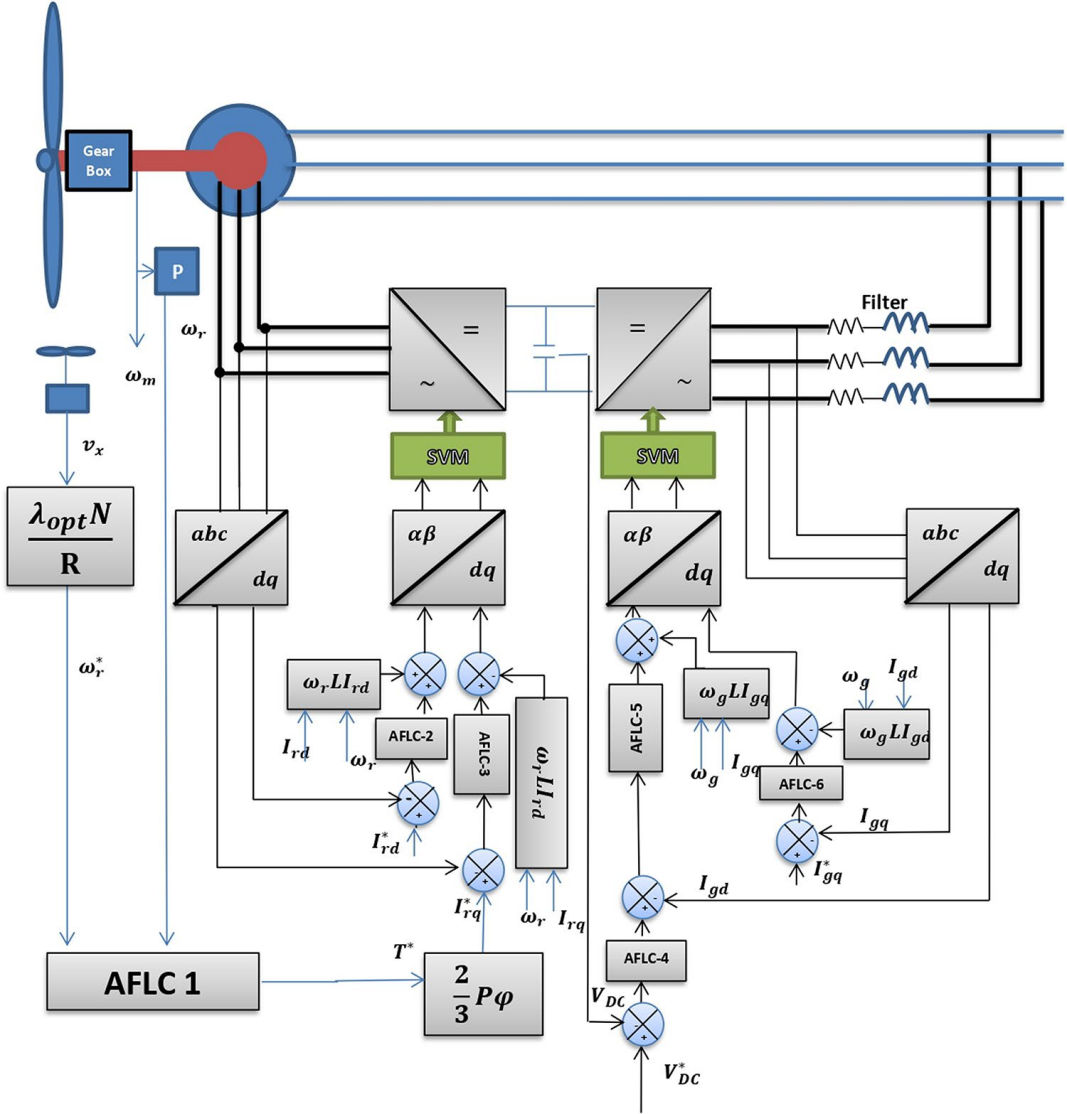
The schematic diagram of DFIG system.

Figure 2 depicts the grid-tied DFIG wind power plant model. The system consists of a VSWT, a DFIG, two variable-speed converters (VSCs), and a DC-bus capacitor.

The WT blades’ mechanical output power P_m_ is illustrated by^9,16,46^

1 $${P}_{m}=\frac{1}{2}\rho A{{v}_{w}}^{3}{C}_{p}(\beta,\lambda)$$

Here is how the ${C}_{p}$ formula is displayed^14^:

2 $$\lambda=\frac{\omega R}{{v}_{w}}$$

3 $${C}_{p}(\beta,\lambda)={D}_{1}\left\{\frac{{D}_{2}}{{\lambda}_{i}}-{D}_{3}\beta-{D}_{4}\right\}{e}^{\frac{{D}_{5}}{{\lambda}_{i}}}{+D}_{6}\lambda$$

4 $$\frac{1}{{\lambda}_{i}}=\frac{1}{\lambda+0.08\beta}-\frac{0.035}{{\beta}^{3}+1}$$

where the wind turbine constants ${D}_{1}$ to ${D}_{6}$ are: ${D}_{1}=0.5176;{D}_{2}=116;{D}_{3}=0.4;{D}_{4}=5;{D}_{5}=-21;{D}_{6}=0.0068;$

**Fig. 3:**
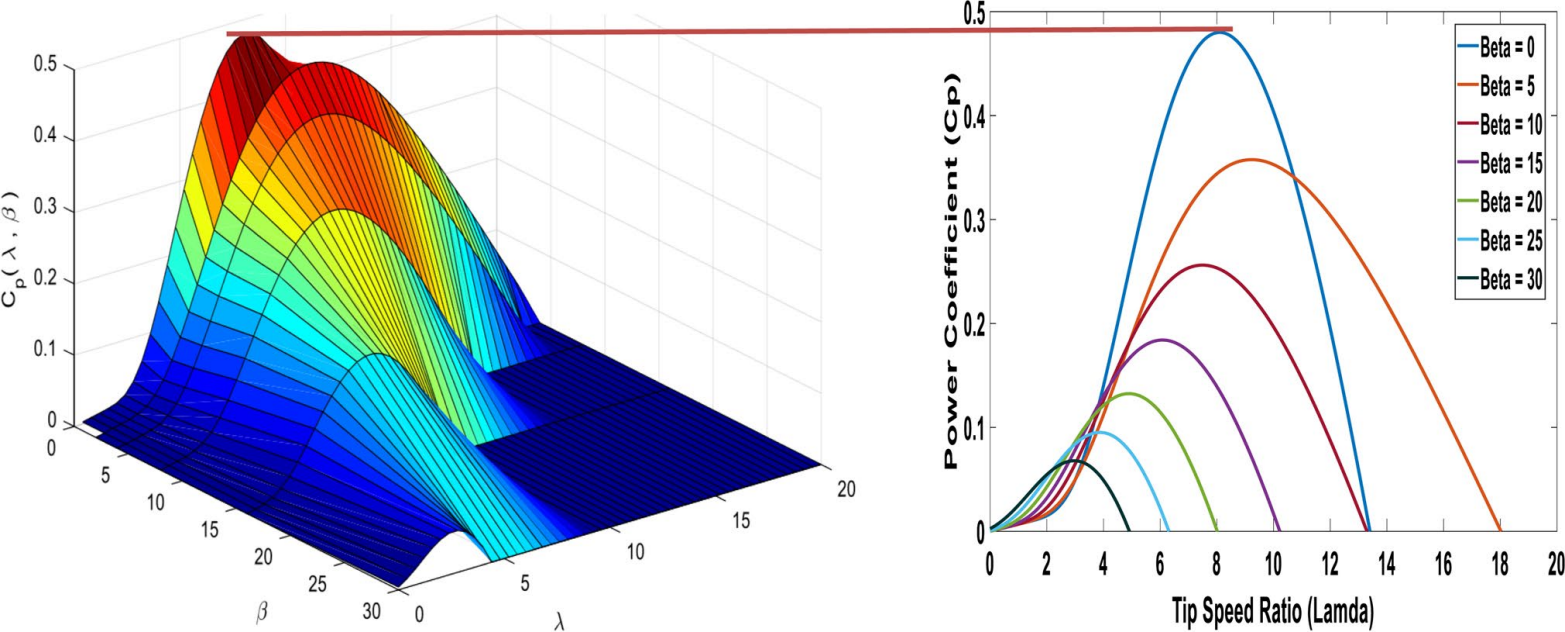
The wind turbine characteristics.

The relationship between the power coefficient (${C}_{p}$ ) and the tip speed ratio ($\lambda$) is illustrated at different pitch angles ($\beta$) in Fig. 3. To achieve the maximum power output, the turbine must operate at the highest ${C}_{p}$, which aligns with the optimal tip speed ratio (${\lambda}_{opt}$).

According to the specifications of the wind turbine, at a pitch angle of $\beta=0$, the optimal tip speed ratio (${\lambda}_{opt}$) is 8.1, and the maximum power coefficient (${{C}_{p}}_{max}$) is 0.48. Therefore, these values correspond to the peak electric power output, as depicted in Fig. 3.

## Adaptive fuzzy logic controller

**Fig. 4:**
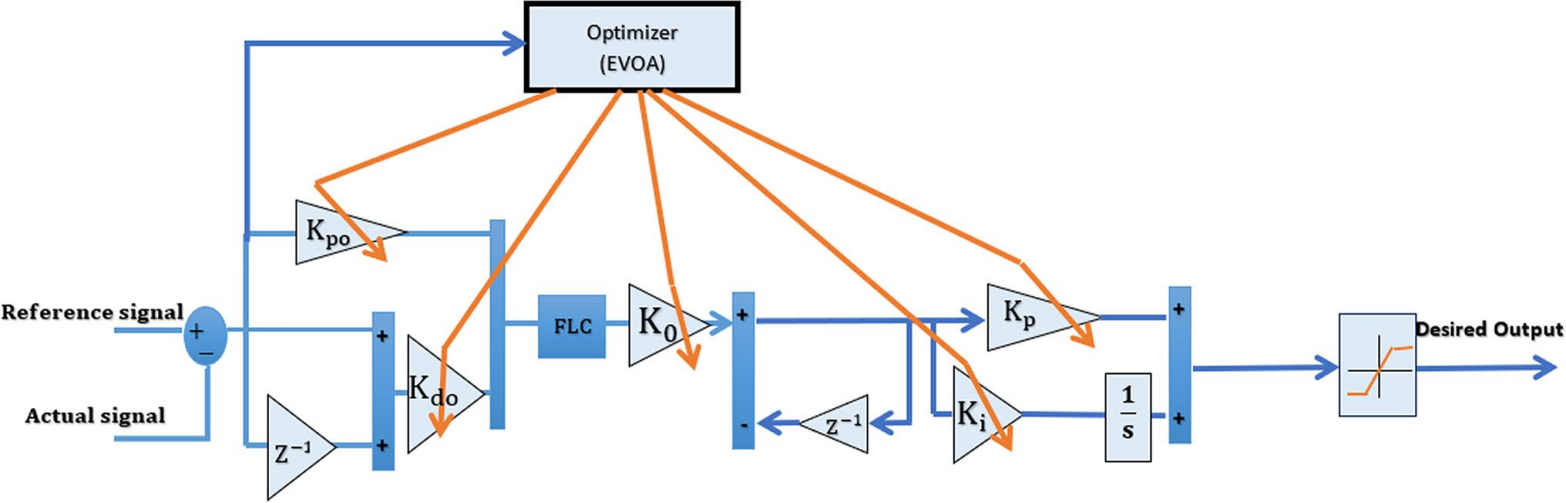
The diagram of the optimal EVOA based AFLC.

The Adaptive Fuzzy Logic Controller (AFLC) architecture includes fuzzification, a fuzzy rule base, and defuzzification components. Figure 4 depicts the optimal AFLCs designed using either the C-BO or EVOA algorithm. This figure illustrates the concept of “adaptive control,” where a fuzzy regulator’s characteristics—such as the fuzzy rules, membership functions, and output scaling factors—can adapt to changes in the system^46^. This adaptability can further reduce rise time. The optimization algorithms (C-BO and EVOA) are employed to fine-tune all parameters (${\text{K}}_{\text{Po}},{\text{K}}_{\text{do}},{\text{K}}_{\text{o}},{\text{K}}_{\text{P}}$ and K_i_) across the six controllers in the system, aiming to minimize the Integrated Squared Error (ISE).

**Fig. 5:**
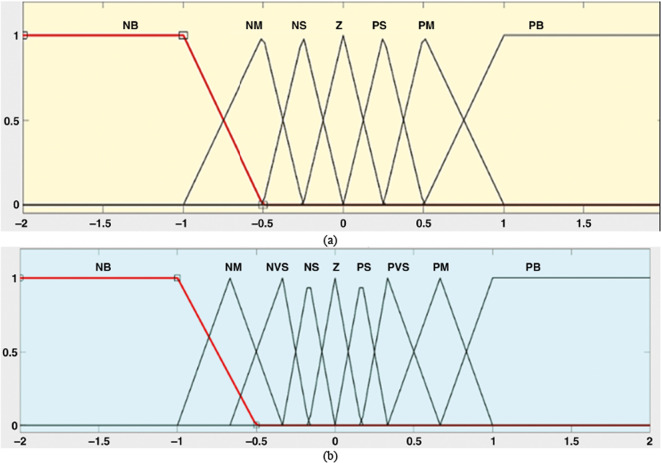
Membership functions; (**a**) for inputs; and (**b**) output.

Additionally, as illustrated in Fig. 5, the linguistic variables are defined as: Positive Big (PB), Positive Big (BB), Positive Big (NB), Positive Big (NM), Positive Big (NS), and Zero (Z) (PB). According to one theory, the triangular MFs with overlap (PB) represent the input/output fuzzy sets^19–21,43^. The fuzzy inference system employs a total of 49 control rules to generate the appropriate signal with the best accuracy and a tolerable computational cost, as detailed in Table 1^9^. It is important to note that the rules are established considering the trade-off between prediction accuracy and the complexity of the Adaptive Fuzzy Logic Controller (AFLC). In an unconstrained situation, increasing the number of fuzzy sets per input variable increases the number of rules firing at once, since each FLC input is fuzzified into a growing number of fuzzy sets, each of which depends on the number of fuzzy sets overlapping each other^9^.

**Table 1:** AFLC rule base.

e	NM	NS	PM	NB	PS	Z	PB
Δe							
NB	NB	NB	NVS	NB	NS	NM	Z
PS	NVS	Z	PM	NS	PS	PVS	PB
NS	NM	NS	PVS	NB	Z	NVS	PS
NM	NB	NM	Z	NB	NVS	NS	PVS
Z	NS	NVS	PS	NM	PVS	Z	PM
PB	PVS	PS	PB	Z	PB	PM	PB
PM	Z	PVS	PB	NVS	PM	PS	PB

## Optimization approaches

### The energy valley optimization approach

EVOA is offered as a revolutionary metaheuristic algorithm that draws inspiration from stability and various particle decay modes seen in advanced physics. The principal idea of the EVOA is based on the fundamental physics principles of the decay process through various particles, therefore the study’s uniqueness may be viewed as inspiring while the test functions’ level of complexity is also being evaluated for the first time in^45^. The initialization technique is carried out in the first stage, where the solution candidates (${\text{Y}}_{\text{i}}$) are particles with varied degrees of stability in the universe (search space), which is considered to represent a special area of the entire search space^45^.

5 $$\text{Y}=\left[\begin{array}{c}{\text{Y}}_{1}\\{\text{Y}}_{2}\\.\\.\\.\\.\\{\text{Y}}_{\text{i}}\\.\\.\\.\\{\text{Y}}_{\text{n}}\end{array}\right]=\left[\begin{array}{c}{\text{y}}_{1}^{1}{\text{Y}}_{1}^{2}..{\text{y}}_{1}^{\text{j}}\dots{\text{y}}_{1}^{\text{d}}\\{\text{y}}_{2}^{1}{\text{y}}_{2}^{2}..{\text{y}}_{2}^{\text{j}}\dots{\text{y}}_{2}^{\text{d}}\\\dots\\\dots\ddots\\\dots\\{\text{y}}_{\text{i}}^{1}{\text{y}}_{\text{i}}^{2}..{\text{y}}_{\text{i}}^{\text{j}}\dots{\text{y}}_{\text{i}}^{\text{d}}\\\dots\\\dots\ddots\\\dots\\{\text{y}}_{\text{n}}^{1}{\text{y}}_{\text{n}}^{2}..{\text{y}}_{\text{n}}^{\text{j}}\dots{\text{y}}_{\text{n}}^{\text{d}}\end{array}\right],\left\{\begin{array}{c}i=1,2,3,.,n\\j=1,2,3,.,d\end{array}\right.$$

6 $${\text{y}}_{\text{i}}^{\text{j}}={\text{y}}_{\text{i,min}}^{\text{j}}+\text{rand}({\text{y}}_{\text{i,max}}^{\text{j}}-{\text{y}}_{\text{i,min}}^{\text{j}})$$

The Enhancement Bound (EB) for the particles is established in the second step of the algorithm, and it is used to compare the properties of neutron-rich and neutron-poor particles. By evaluating each particle’s fitness function, the Neutron Enhancement Level (NEL) of the particles is computed for this purpose. The Enhancement Bound is presented mathematically as following:

7 $$\text{EB}=\frac{\sum_{\text{i}=1}^{\text{n}}{\text{NEL}}_{\text{i}}}{\text{n}},\text{i}=1,2,3\dots\text{n}$$

Based on the evaluations of the objective functions, the the stability degrees ($\text{S}{\text{L}}_{\text{i}}$) of the particles are determined as following:

8 $$\text{S}{\text{L}}_{\text{i}}=\frac{{\text{NEL}}_{\text{i}}-\text{BS}}{\text{WS}-\text{BS}},\text{i}=1,2,3\dots\text{n}$$

The particles WS and BS have the worst and best degrees of stability in the universe, respectively, which correspond to the highest and lowest values of the fitness function.

The process of radioactive decay emitting gamma, beta, or alpha schemes is considered since a particle is presumed to have a higher N/Z ratio in the primary search of the EVOA if its ${\text{NEL}}_{\text{i}}$ exceeds the enrichment bound (EB). In this instance, a random integer between [0, 1] is generated to imitate the Stability Bound (SB) in the universe. In the context of particle decay, it is assumed that alpha or gamma decay will occur if the particle’s stability degree exceeds a certain stability bound ($\text{S}{\text{L}}_{\text{i}}$ > SB). Alpha decays are used to enhance the stability of a product during a process. This results in the creation of a new solution candidate, which can be mathematically represented as one of the position update strategies of the EVOA. Two random numbers are produced for this purpose: Alpha Index I indicates how many rays were released and has integer range of [1, d], and Alpha Index II indicates how many rays were released and has integer range [1, Alpha Index I]. The released rays, which are decision variables in the solution particle (${\text{Y}}_{\text{BS}}$), are replaced by the rays that emanate from the candidate or particle with the highest degree of stability. These elements are expressed mathematically as follows:

9 $${\text{Y}}_{\text{i}}^{\text{n}\text{e}\text{w}1}={\text{Y}}_{\text{i}}\left({\text{Y}}_{\text{B}\text{S}}\right({\text{y}}_{\text{i}}^{\text{j}}\left)\right),\left\{\begin{array}{c}i=1,2,3\dots n\\j=AlphaindexII\end{array}\right.$$

Additionally, the gamma decay process emits gamma rays to increase the stability degree of the excited candidates, hence this element may be mathematically stated as another EVOA position-updating process where a new solution candidate is produced. Gamma Index I indicates the how many photons are released and has a range of [1, d], while Gamma Index II specifies which photons are to be taken into account for calculating the particle’s mass and has a range of [1, Gamma Index I]. Instead of the photons in the particles, which serve as choice variables in the solution candidate and model how excited particles interact with other particles and even magnetic fields, a nearby particle or candidate (${\text{Y}}_{\text{N}\text{g}}$) is used. To calculate the overall distance between the particle under examination and the other particles in this situation, the nearest particle is used:

10 $${D}_{i}^{k}=\sqrt{{\left({y}_{2}-{y}_{1}\right)}^{2}+{\left({z}_{2}-{z}_{1}\right)}^{2}},\left\{\begin{array}{c}i=1,2,3,.,n\\k=1,2,3,.,n-1\end{array}\right.$$

where (${y}_{1}$, ${z}_{1}$ ) as well as (${y}_{2}$, ${z}_{2}$ ) are the particles coordinates in the universe.

The following activities are used in the position update process to create the second solution candidate in this step:

11 $${\text{Y}}_{\text{i}}^{\text{n}\text{e}\text{w}2}={\text{Y}}_{\text{i}}\left({\text{Y}}_{Ng}\right({\text{y}}_{\text{i}}^{\text{j}}\left)\right),\left\{\begin{array}{c}i=1,2,3\dots n\\j=AlphaindexII\end{array}\right.$$

Because beta decay occurs in particle with high level of instability, it is assumed to occur when a particle’s stability degree is lower than the stability bound ($S{L}_{i}\le SB$). Due to the greater degrees of instability in these particles, a large leap in the search space is expected to occur in the scenario of beta decay, where beta are ejected from the particles to enhance their degree of stability. In this case, the particles go through a process of updating their positions where a controlled movement is made towards the candidate or particle with the optimum stability degree (${Y}_{BS}$) and the particles’ centre (${Y}_{CP}$). These algorithmic features imitate the candidate s’ propensity to approach the band of stability, where the majority of known particles are located and where the majority of them have higher degrees of stability. These elements are expressed mathematically as follows:

12 $${\text{Y}}_{\text{C}\text{P}}=\frac{\sum_{\text{i}=1}^{\text{n}}{\text{Y}}_{\text{i}}}{\text{n}},\text{i}=1,2,3\dots\text{n}$$

$${Y}_{i}^{new1}={Y}_{i}+\frac{(ran{d}_{1}x{Y}_{BS}-ran{d}_{2}x{Y}_{CP})}{S{L}_{i}},\text{i}=1,2,3\dots\text{n}$$

For the particles employing beta decay, a different position updating procedure is carried out where a controlled movement is performed towards the candidate with the optimal stability degree (${Y}_{BS}$) and a nearby candidate (${Y}_{Ng}$), while the stability degree of the particle has no bearing on the process of movement. These elements are expressed as following:

13 $${Y}_{i}^{new2}={Y}_{i}+(ran{d}_{3}x{Y}_{BS}-ran{d}_{4}x{Y}_{Ng}),$$

$$\text{i}=1,2,3\dots\text{n}$$

A candidate is thought to have a lower $\text{N}/\text{Z}$ ratio if its neutron enrichment level is less than or equal the enrichment bound ($\text{N}\text{E}{\text{L}}_{\text{i}}\le\text{E}\text{B}$). As a result, the particle is more likely to travel towards the stability band by electron capture or positron emission. In order to take these kinds of movements into consideration, a random movement in the search space is chosen as follows:

14 $${Y}_{i}^{new}={Y}_{i}+rand,\text{i}=1,2,3\dots\text{n}$$

**Fig. 6:**
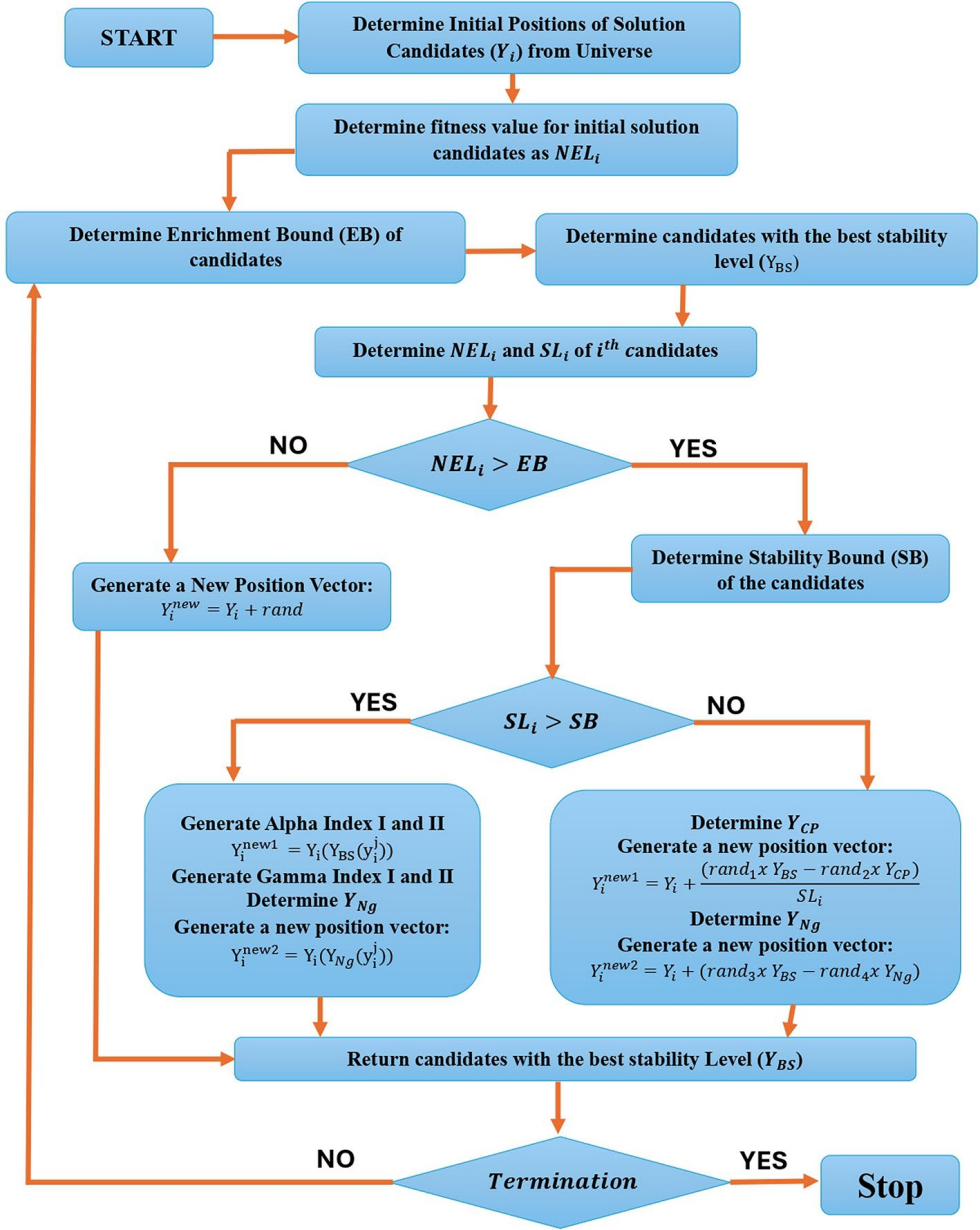
EVOA flowchart.

Only two freshly formed position vectors, ${\text{Y}}_{\text{i}}^{\text{n}\text{e}\text{w}1}$ and ${\text{Y}}_{\text{i}}^{\text{n}\text{e}\text{w}2}$, are generated for each particle if its enrichment level exceeds $\text{E}\text{B}$; for particles with lower enrichment levels, only ${\text{Y}}_{\text{i}}^{\text{n}\text{e}\text{w}}$ is produced. The freshly created vectors are combined with the existing population at each state, and the top particles then take part in the algorithm’s next search loop. For decision variables that exceed the upper and lower boundaries, a boundary violation flag is set, and as a termination criterion. Figure 6 illustrates the flowchart of EVOA.

### The C-BO approach

The C-BO is a new optimizer approach introduced in 2021^47^, based on the game of billiards^48^. In this game, balls are pushed around a table with six pockets using a cue. The balls are initially placed at random and some of the best are chosen for pockets. The BOA technique starts with a random selection of agents, making it difficult to establish a precise initialization strategy. Combining meta-heuristic (BOA) and chaotic algorithms can improve performance, because logistic maps is a good choice for faster local searches^49–51^.

### Genetic approach

**Fig. 7:**
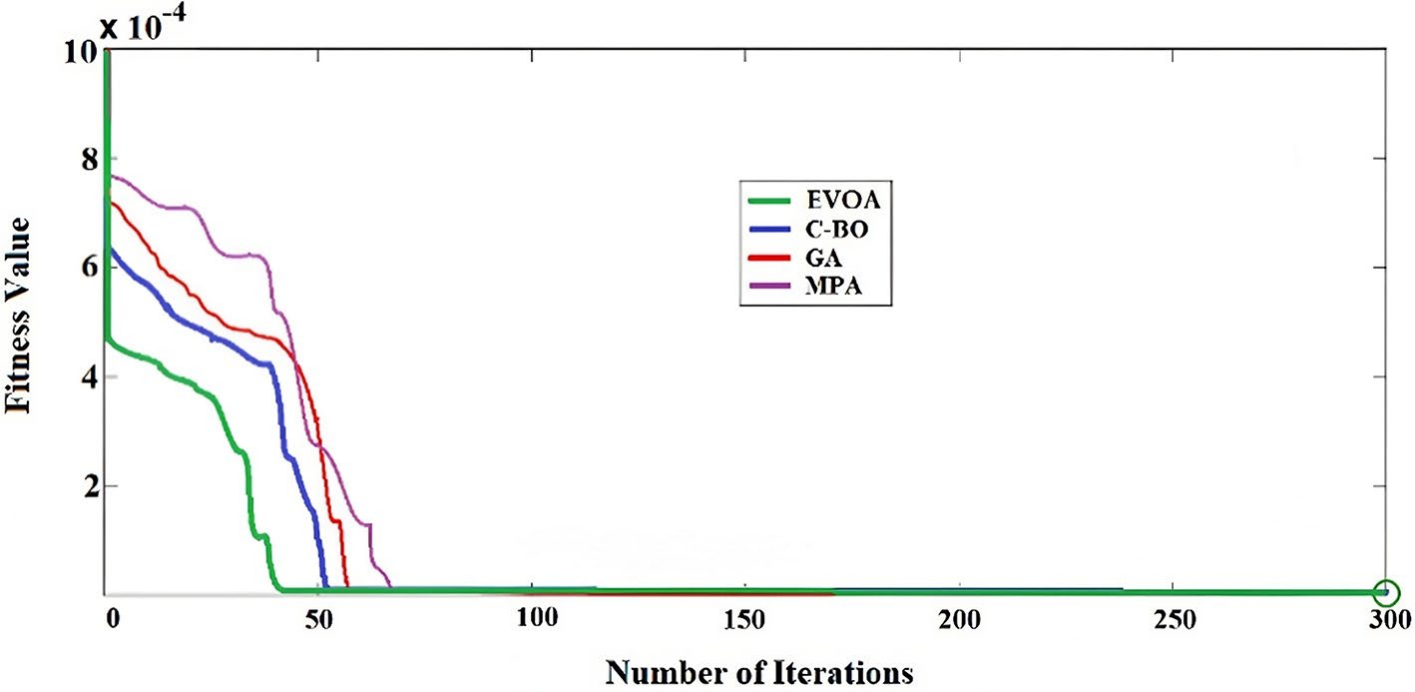
The fitness functions’ convergence.

The genetic algorithm (GA) emulates natural evolutionary processes to identify optimal solutions for various engineering challenges^33,52,53^. It operates on a population of chromosomes representing potential solutions, evaluating each based-on fitness. The GA employs techniques such as selection, reproduction, mutation, and crossover to generate new solutions and enhance the population. By adhering to the “survival of the fittest” principle, the GA progressively converges toward the best solution^54,55^. Figure 7 demonstrates the fitness functions (ISE of all six controllers) convergence rate for EVOA, C-BO, GA, and MPA, and compared under identical conditions, showing minimal changes. The GA parameters used in this study are detailed in Table 2. Notably, the C-BO-AFLC and EVOA-AFLC algorithms converge more quickly compared to the GA-AFLC and MPC-PI. The optimal gains (${\text{K}}_{\text{P}\text{o}},{\text{K}}_{\text{d}\text{o}},{\text{K}}_{\text{o}},{\text{K}}_{\text{P}}\text{a}\text{n}\text{d}{\text{K}}_{\text{i}}$) for the six controllers of AFLCs are outlined in Table 3. The optimal gains (${\text{K}}_{\text{P}}\text{a}\text{n}\text{d}{\text{K}}_{\text{i}}$) for the six controllers of PIs using MPA are outlined in Table 4 to produce the minimum value of ISE. Since C-BO and EVOA are stochastic optimization methods, 40 independent optimization runs are conducted to determine the statistical measurements as the mean, standard deviation, and the required number of objective function evaluations. A predetermined stopping criterion is also taken into consideration, which is based on a tolerance of 1 × 10^− 12^ for the global best values of the claimed problem and a maximum of 100,000 objective function evaluations. Table 5 presents the minimum, maximum, mean, standard deviation (SD), and the required number of evaluations of the optimized objective function (ISE) from forty separate runs, demonstrating that EVOA consistently performs well. MPA and GA required an average of 100,000 objective function evaluations while C-BO with 93,117.2, and EVOA with 78,431.2 have better performance. The low standard deviations associated with EVOA highlight its stability, with the algorithm achieving the optimal solution in just 8 s using the minimum ISE. The main constraints facing the optimization are the stator machine current must be below the rated current of the machine and mechanical power produced by DFIG must be below the rated power. AFLC such any controller with integral part has a problem with windup we have overcome this problem by working in MPPT region (from cut in speed and rated wind speed).

**Table 2:** Genetic parameters.

Fitness scaling	Crossover		Demographics		Migration fraction	Migration interval
	Function	Fraction	Type	Size		
Rank	Scattered	0.9	Double Vector	60	0.25	15

**Table 3:** Optimum parameters for AFLC regulators.

Algorithm	${k}_{p0}$	${k}_{do}$	${k}_{0}$	$kp$	$ki$
*C-BO*					
AFLC-1	10.759	4.589	0.1563	9.756	4.659
AFLC-2	5.236	1.549	2.258	5.214	1.248
AFLC-3	1.688	2.516	0.312	8.345	0.2789
AFLC-4	8.146	0.2789	0.9815	1.6345	0.544
AFLC-5	0.177	5.8721	1.246	0.0023	6.234
AFLC-6	8.012	0.0154	2.4902	6.567	0.002
*EVOA*					
AFLC-1	15.269	6.698	1.538	10.268	5.263
AFLC-2	6.236	1.869	3.689	7.456	1.528
AFLC-3	1.556	3.681	0.254	9.355	0.4569
AFLC-4	9.232	0.3892	1.003	2.7455	0.664
AFLC-5	0.223	6.728	1.756	0.0235	8.365
AFLC-6	9.546	0.0254	3.523	9.563	0.0001
*GA*					
AFLC-1	5.255	1.654	0.0263	5.125	3.647
AFLC-2	4.632	1.325	2.121	4.019	2.694
AFLC-3	3.742	1.965	0.222	6.453	0.258
AFLC-4	6.658	0.299	0.9623	0.936	0.825
AFLC-5	0.0451	4.325	1.003	0.12	4.263
AFLC-6	10.596	0.0001	1.9235	4.572	0.226
*MPA*					
AFLC-1	10.256	5.293	0.0362	5.526	2.932
AFLC-2	1.236	1.253	2.563	4.529	3.269
AFLC-3	2.253	2.568	0.523	4.253	0.215
AFLC-4	8.893	0.356	0.8612	0.853	0.816
AFLC-5	0.0326	5.269	1.505	0.135	3.263

**Table 4:** Optimum parameters for PI regulators.

Algorithms	K_p._	K_i_
*MPA*		
PI-1	9.03	4.283
PI-2	4.185	1.581
PI-3	6.984	3.91
PI-4	5.060	1.628
PI-5	0.89	2.013
PI-6	3.287	1.256

**Table 5:** Statistical analysis for the fitness function using EVOA-AFLC, C-BO-AFLC, GA-AFLC, and MPA-PI algorithms.

Technique	Max.	Min.	Mean	Std. dev.	Objective function evaluations	Computational time
PI using MPA	0.0982	0.0515	0.0745	0.0235	100,000	11.3
AFLC using GA	0.0934	0.0503	0.0745	0.0235	100,000	10.2
AFLC using C-BO	0.0436	0.0324	0.036	0.0036	93,117.2	9.6
AFLC using EVOA	0.0319	0.0261	0.0289	0.0014	78.431.2	8.0

## Simulation results and discussion

In this research, a grid-connected DFIG wind power plant model was developed using the MATLAB/Simulink environment. A step change in wind speed was used to test the durability of the optimized control and evaluate the performance of several adaptive fuzzy logic controllers (AFLCs) optimized by the Energy Valley Optimizer Algorithm (EVOA), comparing it to controllers based on the Chaotic-Billiards Optimization Algorithm (C-BO-AFLC), Genetic Algorithm (GA-AFLC), and traditional PI controllers. System parameters are presented in Table 6.

**Table 6:** System parameters.

Nominal Power	P_nom_ = 1650 kw
Voltage	${\text{V}}_{\text{n}\text{o}\text{m}}=575\text{V}$
Frequency	$\text{f}=50\text{H}\text{z}$
Stator Leakage inductance	${\text{L}}_{\text{s}}=0.171\text{p}\text{u}$
Rotor Leakage inductance	${\text{L}}_{\text{r}}=0.1560\text{p}\text{u}$
Stator Resistance	${\text{R}}_{\text{s}}=0.0071\text{p}\text{u}$
Rotor Resistance	${\text{R}}_{\text{r}}=0.0050\text{p}\text{u}$
Magnetization inductance	${\text{L}}_{\text{m}}=2.9\text{p}\text{u}$
No. of Pair poles	$\text{p}=3$

### Case I: Step wind profile

**Fig. 8:**
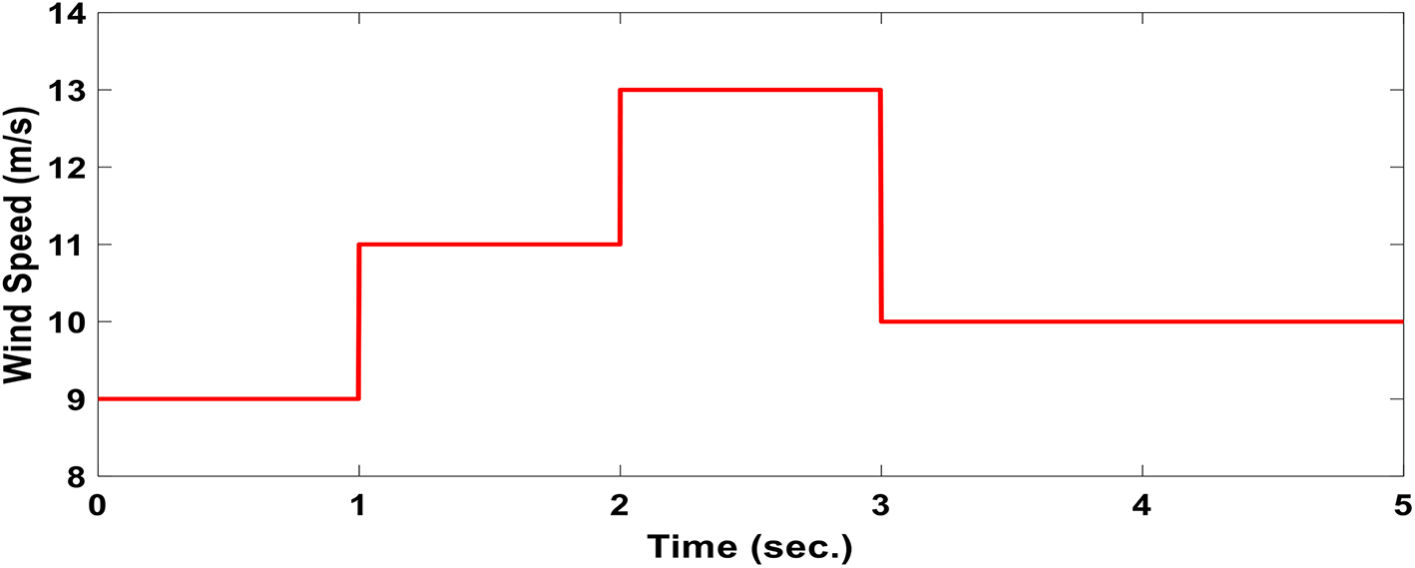
Wind speed profile.

**Fig. 9:**
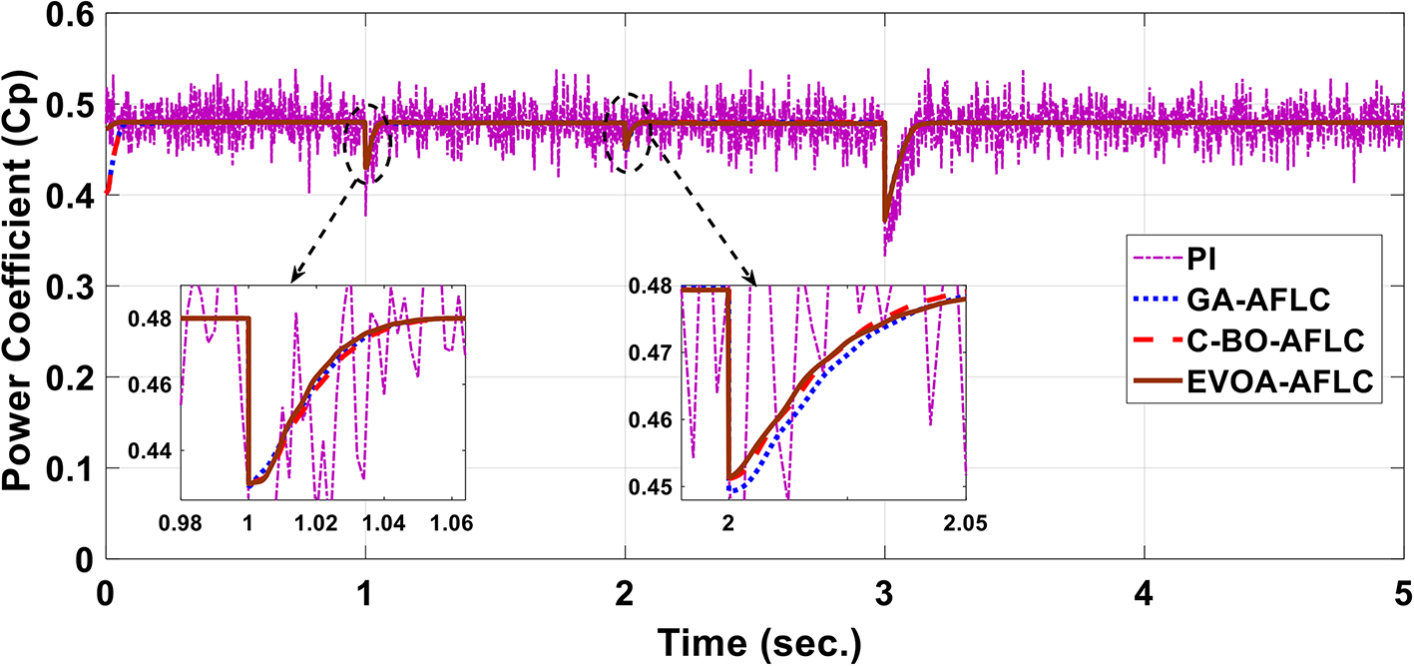
Power coefficient.

**Fig. 10:**
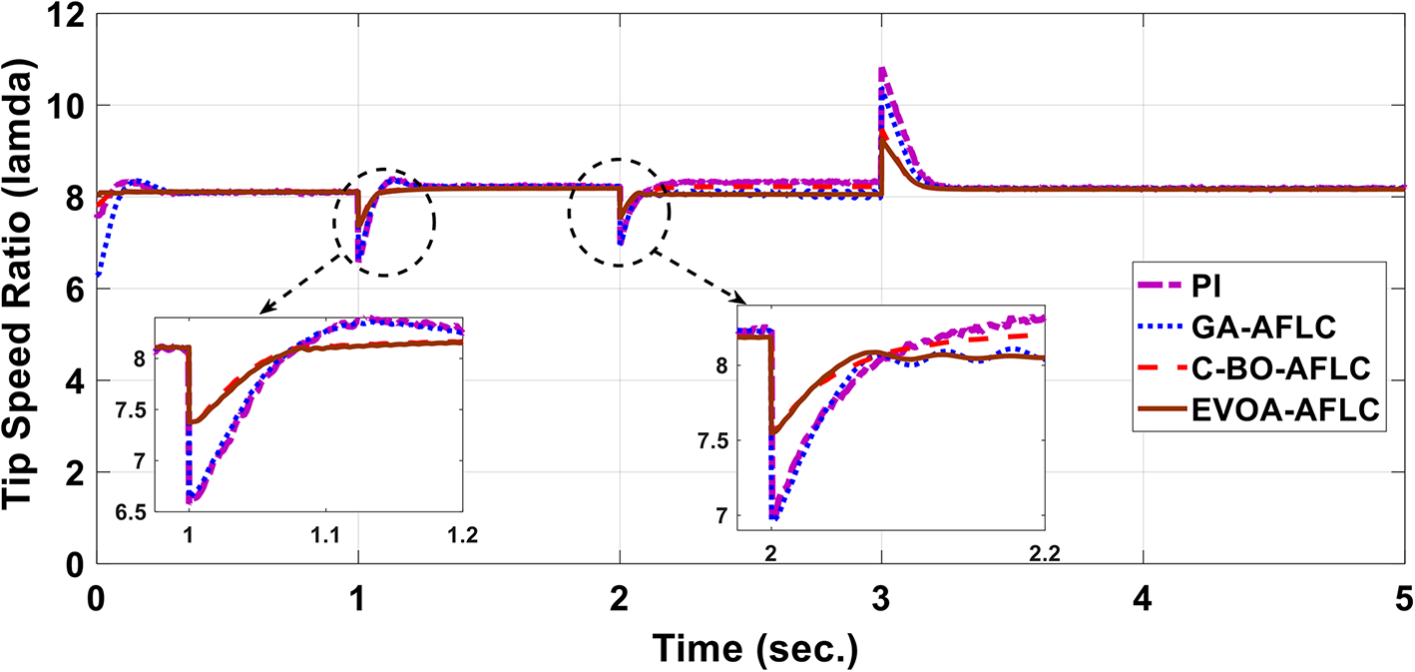
Tip speed ratio.

**Fig. 11:**
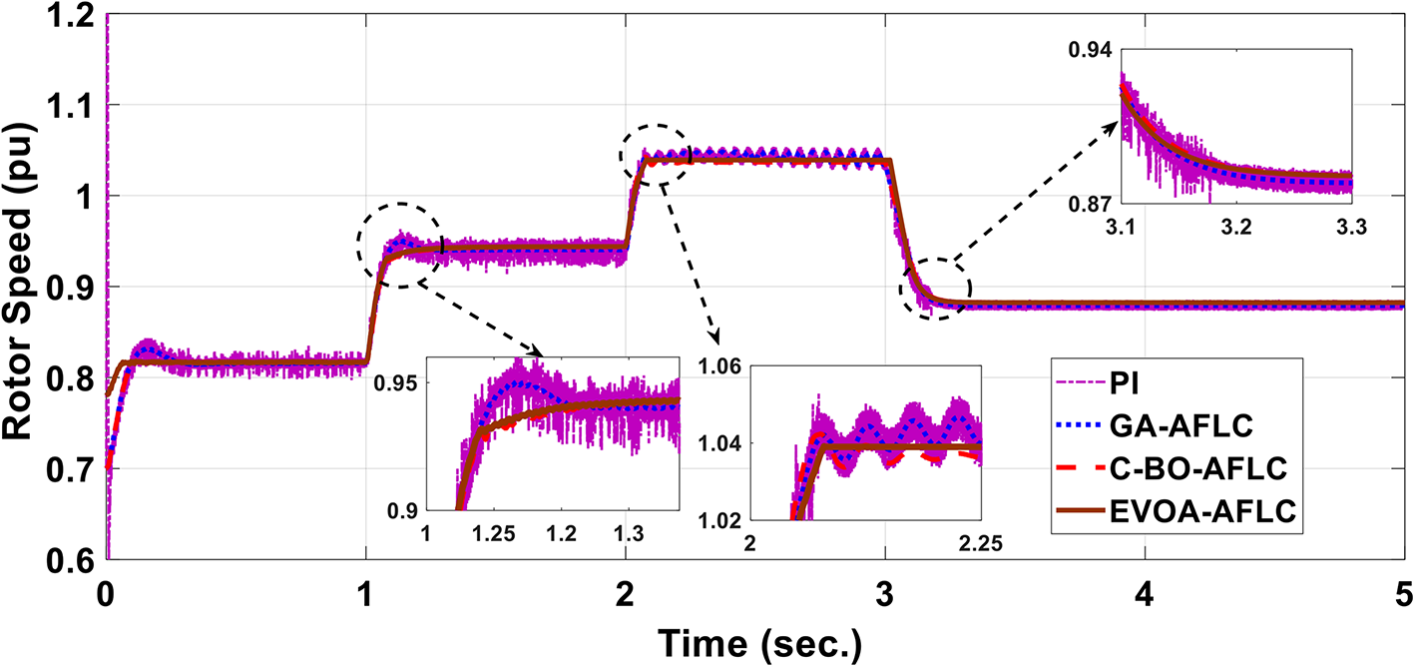
Mechanical rotor speed.

**Fig. 12:**
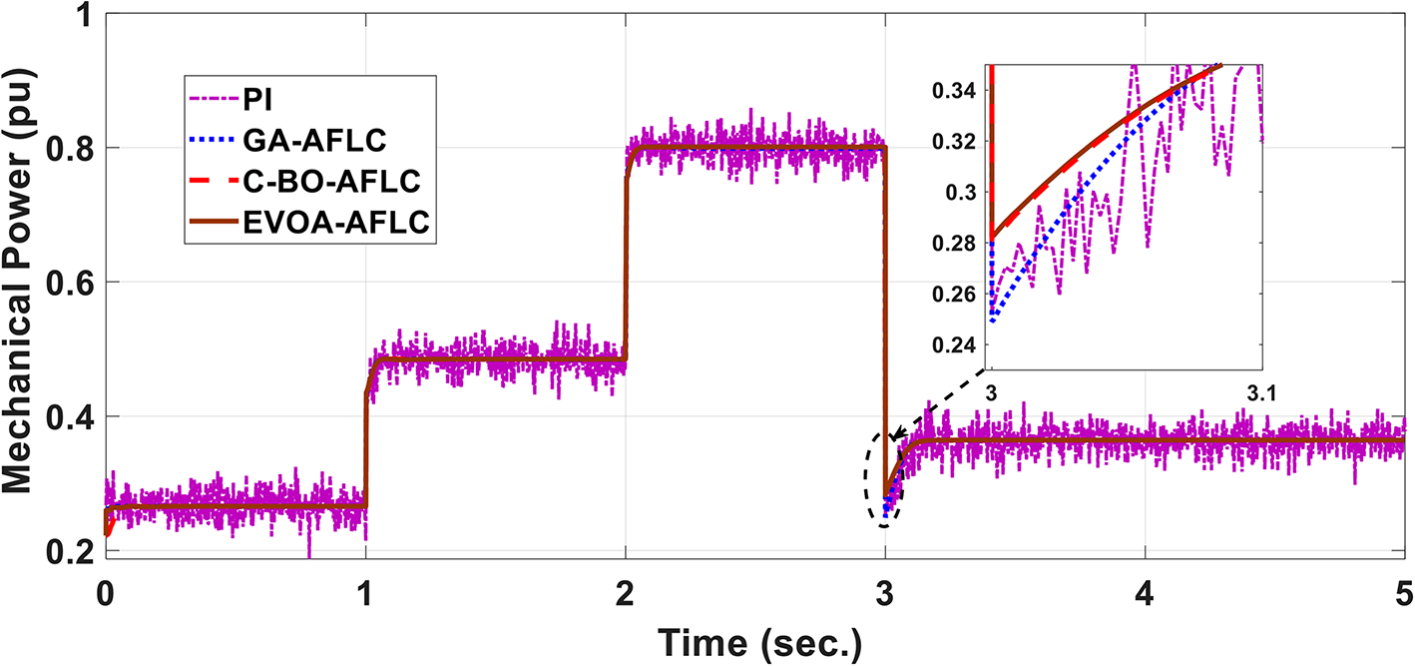
Mechanical power.

**Fig. 13:**
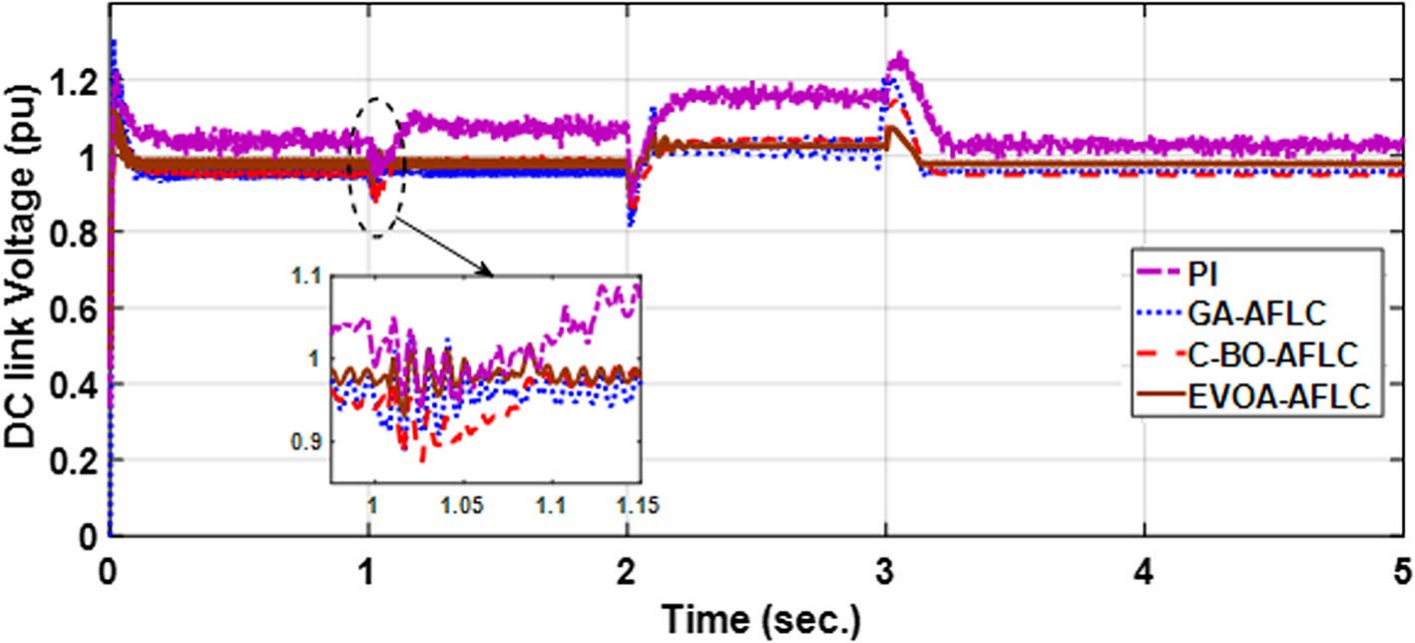
DC link voltage wave form.

**Fig. 14:**
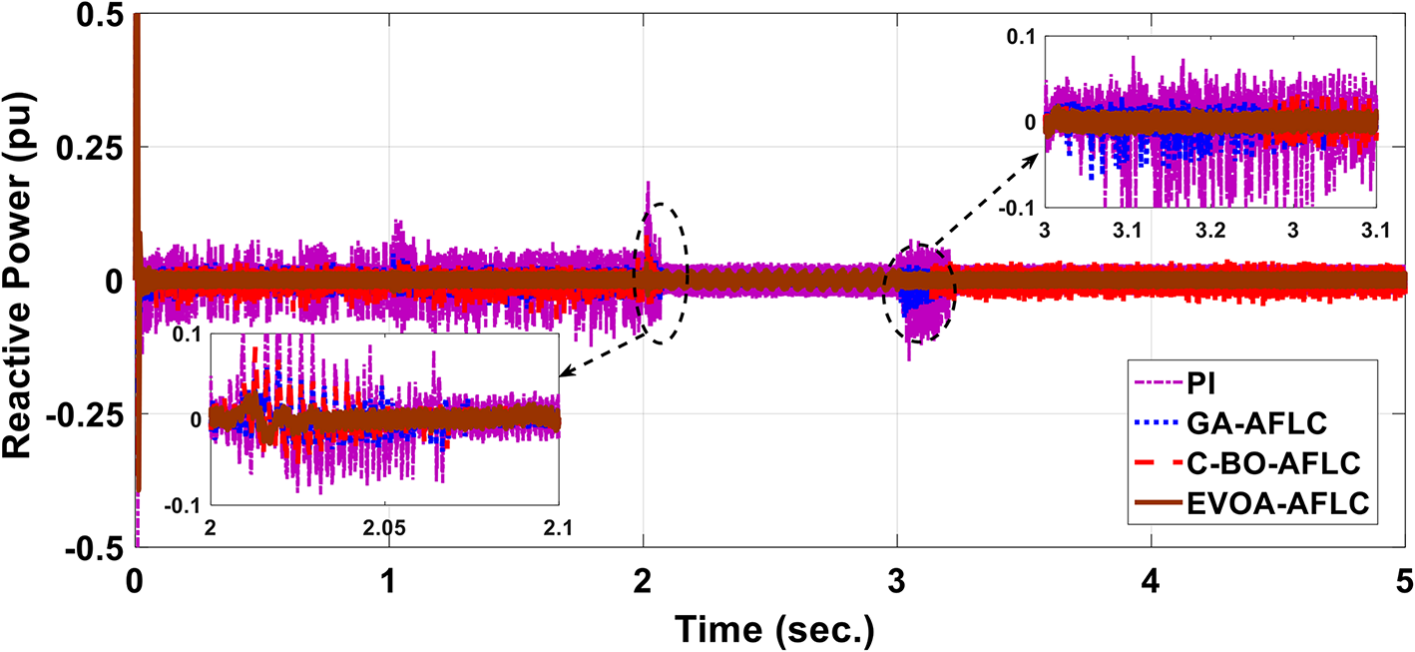
Grid reactive power waveform out of DFIG.

The wind speed profile, which steps over a 5-second period, is shown in Fig. 8. As depicted in Fig. 9, the power coefficient (Cp) was optimized to a peak value of 0.48 by employing the Rotor Side Converter (RSC), ensuring that the controllers worked at maximum efficiency. Both the EVOA-AFLC and C-BO-AFLC demonstrated superior speed of response and lower undershoot at t = 1s and t = 2s when compared to the GA-AFLC and MPA-PI controllers. Among them, the EVOA-AFLC slightly outperformed the C-BO-AFLC. As seen in Fig. 10, the Tip Speed Ratio (TSR) is maintained at 8.1, with the EVOA-AFLC and C-BO-AFLC delivering quicker TSR adjustments and achieving the desired ratio with minimal steady-state error. Notably, the EVOA-AFLC had the lowest peak undershoot at t = 3s and experienced less overshoot at t = 1s and t = 2s. Figure 11 highlights the effectiveness of the EVOA-AFLC and C-BO-AFLC in Maximum Power Point Tracking (MPPT), with the DFIG closely following the rotor speed reference at t = 1s, t = 2s, and t = 3s. The EVOA-AFLC displayed marginally better tracking than the C-BO-AFLC. Conversely, the GA-AFLC and MPA-PI controllers exhibited the highest steady-state errors between t = 2s and t = 3s. The EVOA-AFLC enhanced speed tracking by 86.3% compared to the MPA-PI, 56.36% over the GA-AFLC, and 39.3% compared to the C-BO-AFLC, all while minimizing peak overshoots at t = 1s, t = 2s, and t = 3s. Figure 12 demonstrates the mechanical power output from the wind turbine. At t = 3s, the C-BO-AFLC and EVOA-AFLC reached undershoot values of 0.287pu and 0.29pu respectively, outperforming the GA-AFLC (0.24pu) and MPA-PI (0.238pu). In Fig. 13, the DC link voltage for each control approach is shown, with the EVOA-based AFLC offering the fastest stabilization and the least steady-state error, effectively handling disturbances at t = 2s and t = 3s. Finally, Fig. 14 shows the reactive power waveforms for the tested control techniques, maintaining the reference value of ${Q}_{ref}=0$ Var. The EVOA-AFLC again exhibited improved performance, with lower overshoot, minimal undershoot, and better damping characteristics when compared to the C-BO-AFLC, MPA-PI, and GA-AFLC.

**Fig. 15:**
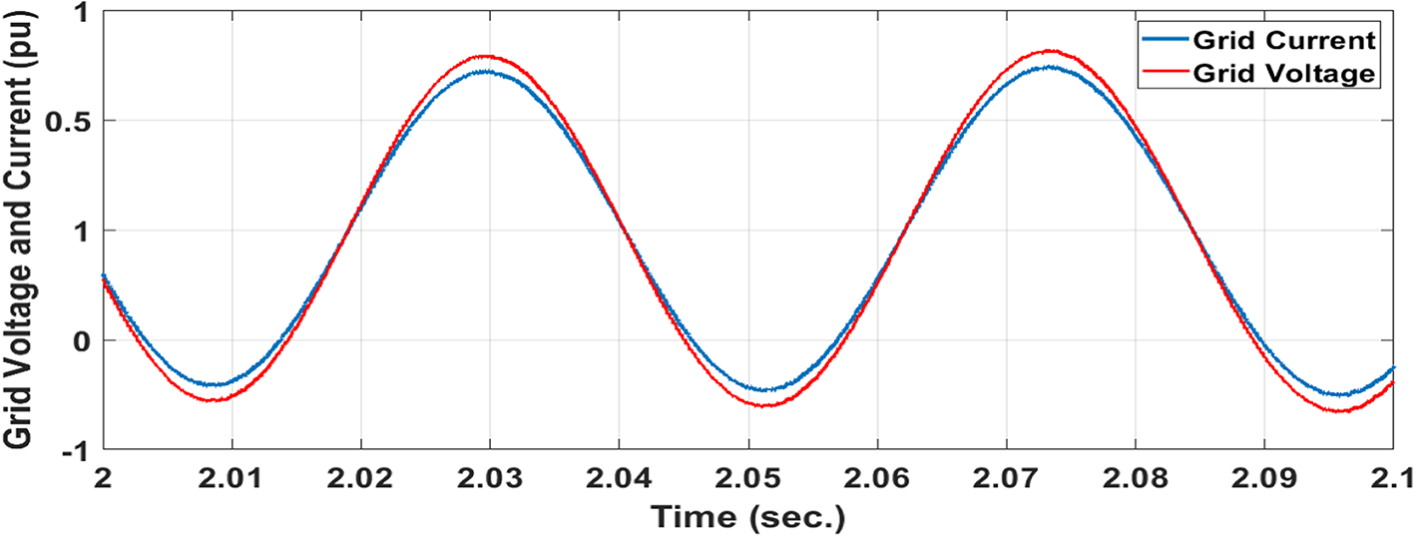
Grid current and voltage using EVOA-AFLC scheme.

In Fig. 15, the grid’s voltage and current are in phase, indicating the GSC controller’s good reaction when employing EVOA-AFLC. Power factor operation is attained to unity as a result.

### Case II: Real wind profile

**Fig. 16:**
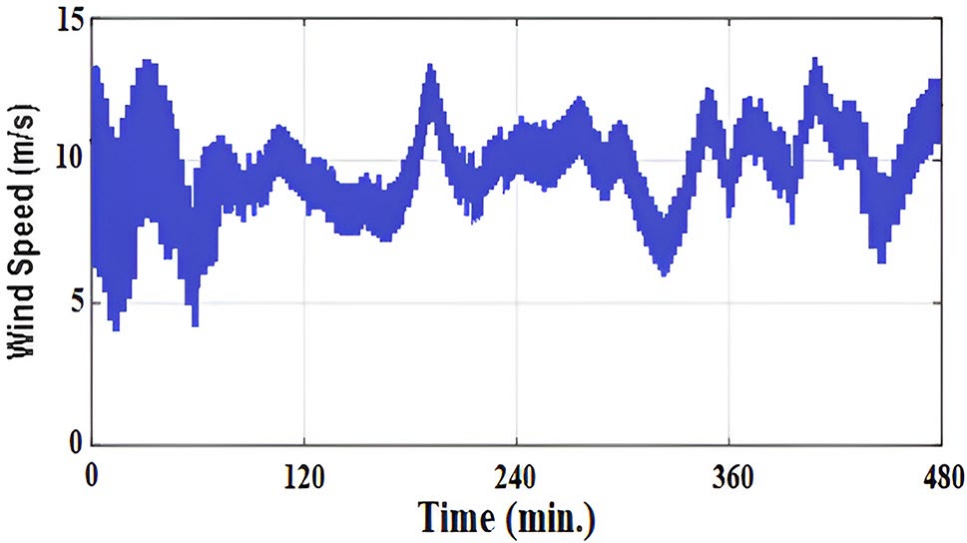
Realistic wind speed profile.

**Fig. 17:**
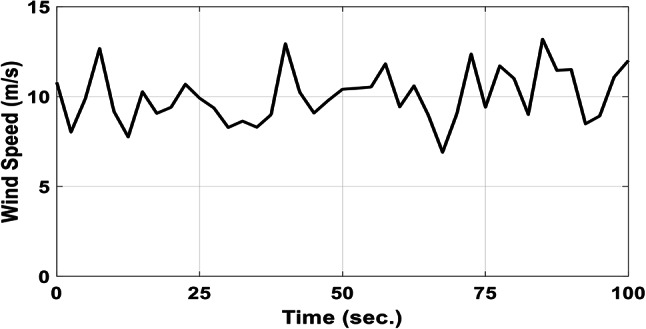
Wind speed profile.

**Fig. 18:**
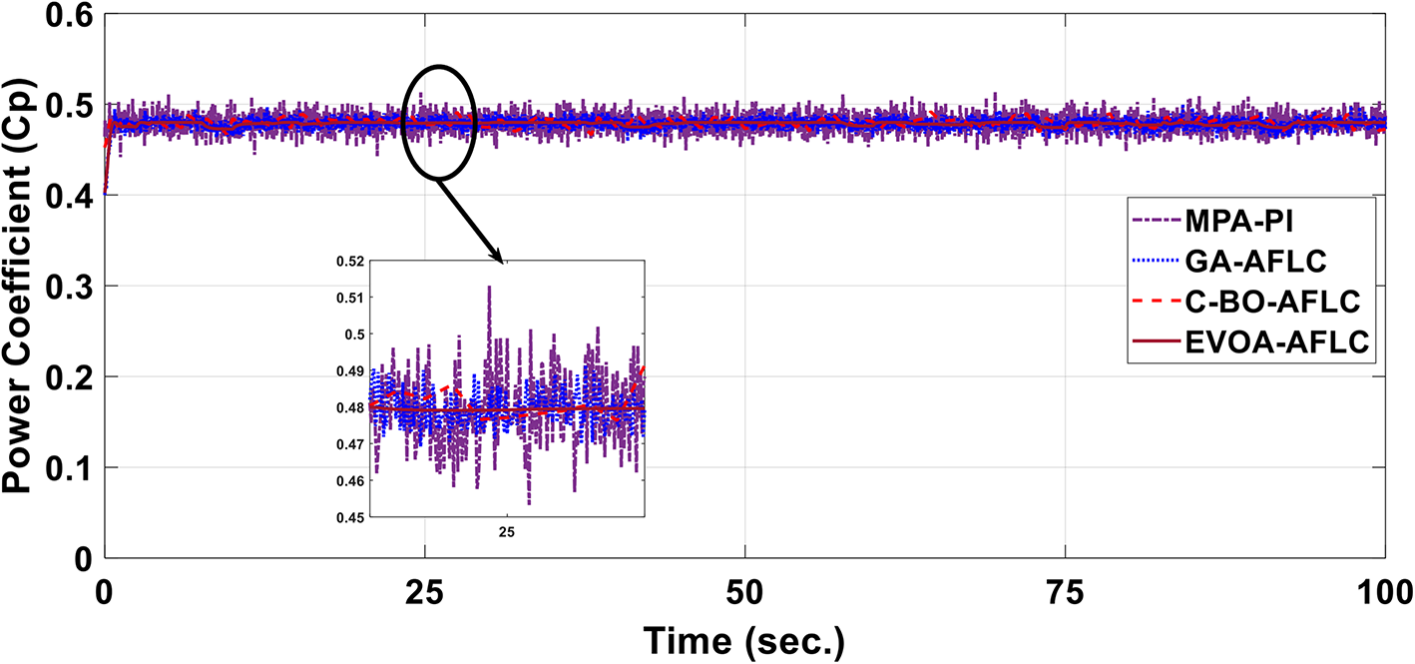
Power coefficient.

**Fig. 19:**
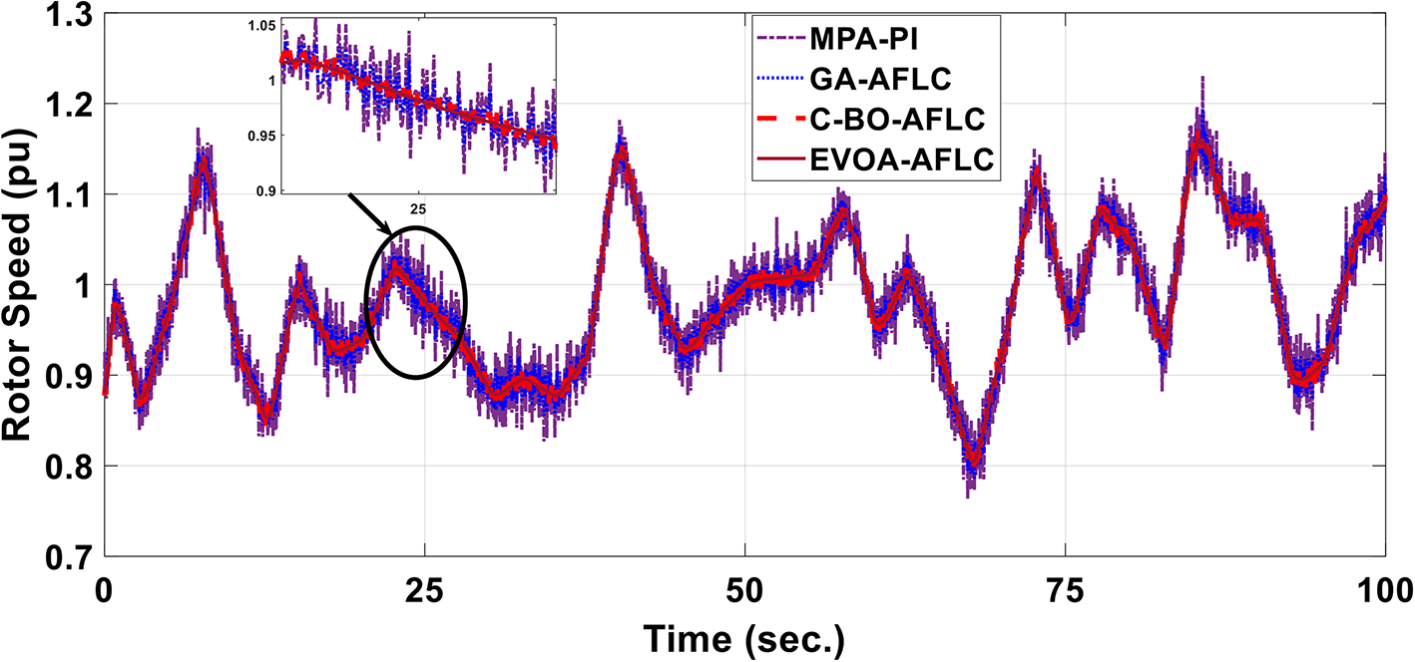
Mechanical rotor speed.

**Fig. 20:**
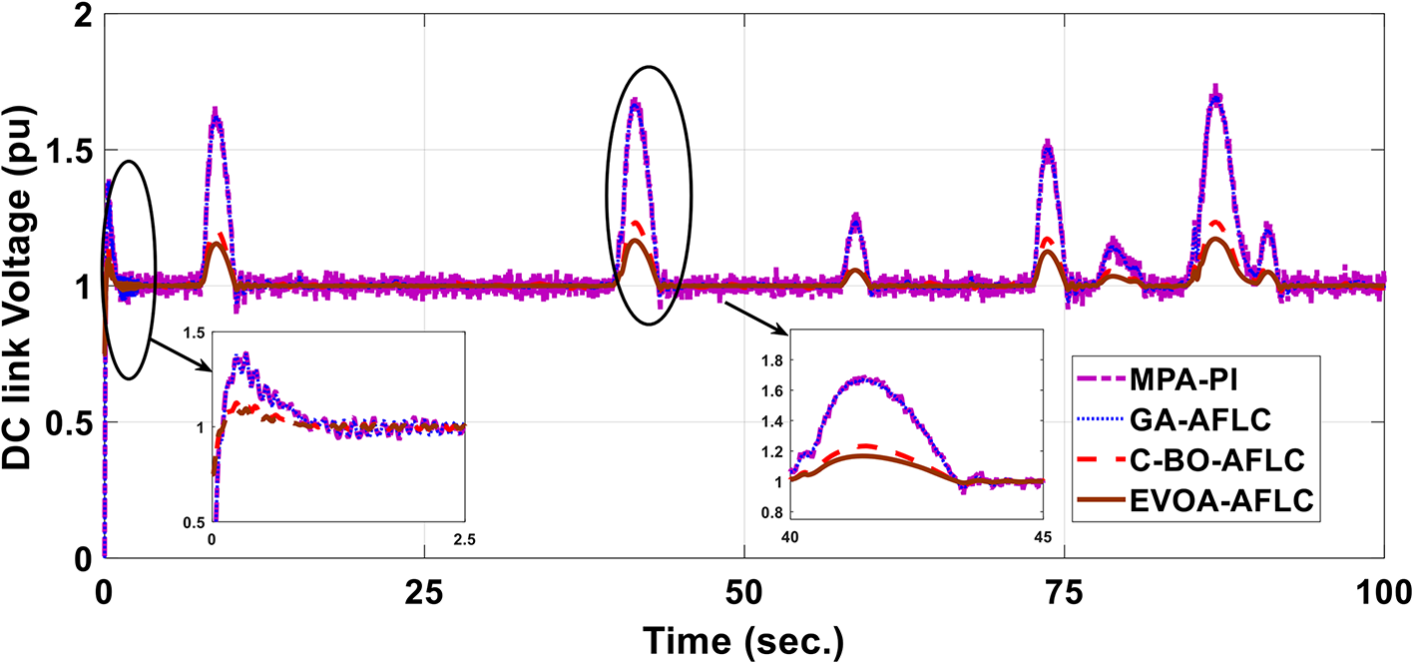
DC link voltage wave form.

**Fig. 21:**
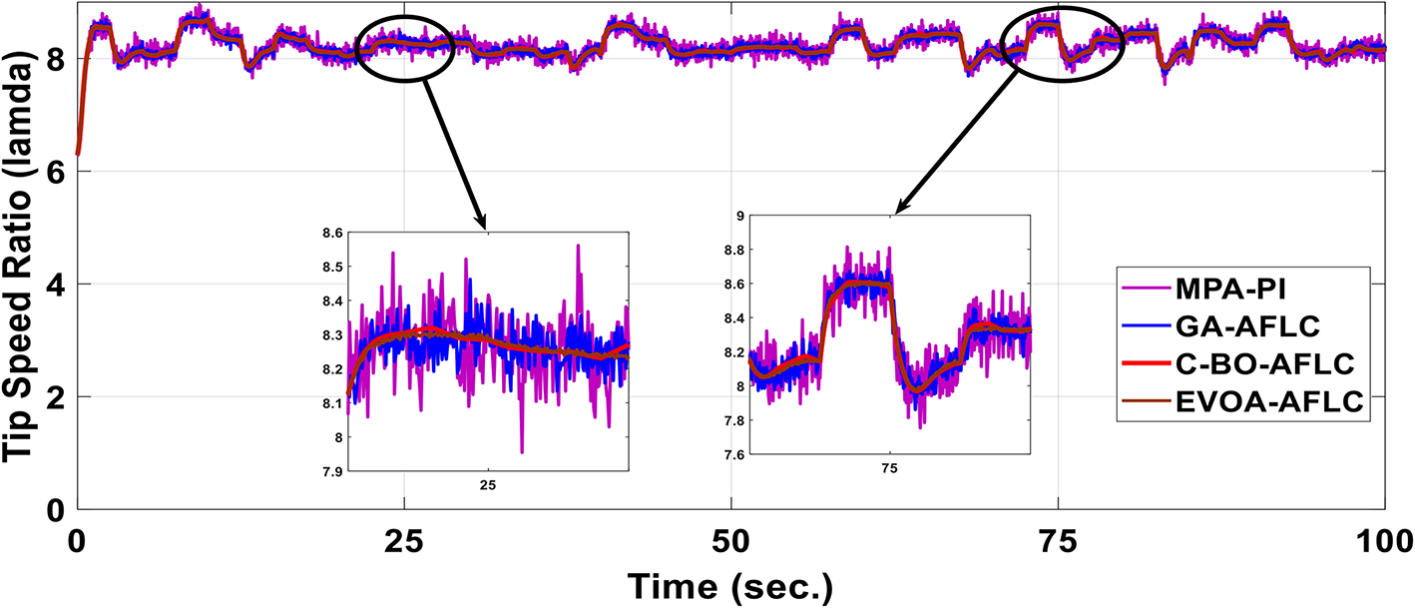
Tip speed ratio.

A realistic wind speed profile has been harvested from Ras Gareb Egypt as shown in Fig. 16. The average wind speed varies from 7.5 m/s in Summer to 15 m/s in Winter. The wind speed profile is rescaled and reprocessed for 100 s. as shown in Fig. 17 to fit the simulation. As depicted in Fig. 18, the power coefficient (Cp) was optimized to a peak value of 0.48 by employing the Rotor Side Converter (RSC), ensuring that the controllers worked at maximum efficiency. Both the EVOA-AFLC and C-BO-AFLC demonstrated superior speed of response and lower undershoot when compared to the GA-AFLC and MPA-PI controllers. Among them, the EVOA-AFLC slightly outperformed the C-BO-AFLC. Figure 19 highlights the effectiveness of the EVOA-AFLC and C-BO-AFLC in Maximum Power Point Tracking (MPPT), with the DFIG closely following the rotor speed reference. The EVOA-AFLC displayed marginally better tracking than the C-BO-AFLC. Conversely, the GA-AFLC and MPA-PI controllers exhibited the highest steady-state errors. The EVOA-AFLC enhanced speed tracking by 73.6% compared to the MPA-PI, 42.28% over the GA-AFLC, and 31.25% compared to the C-BO-AFLC, all while minimizing peak overshoots. In Fig. 20, the DC link voltage for each control approach is shown, with the EVOA-based AFLC offering the fastest stabilization and the least steady-state error, effectively handling disturbances. As seen in Fig. 21, the Tip Speed Ratio (TSR) is maintained at 8.1, with the EVOA-AFLC and C-BO-AFLC delivering quicker TSR adjustments and achieving the desired ratio with minimal steady-state error. Notably, the EVOA-AFLC had the lowest peak undershoot at t = 75s and experienced less overshoot.

The results clearly demonstrate that the EVOA-AFLC delivers enhanced performance in terms of maximum power tracking and restoring system stability. Additionally, an analysis of the integral absolute error (IAE) for system evaluation, as given by the following equation, further emphasizes the effectiveness of the EVOA-AFLC:

15 $$IAE={\int}_{0}^{\infty}\left|e\left(t\right)\right|dt$$

Tables 7 and 8 provide the tracking error values for each AFLC, showing that the EVOA-based controller consistently achieved the smallest error. Consequently, the MPPT results with EVOA represent the optimal solution. The EVOA-AFLC reduced the mean square error by 71.2% compared to the GA-AFLC, 24.4% compared to the C-BO-AFLC, and 84% compared to the MPA-PI.

**Table 7:** IAE values for the GA-AFLC, C-BO-AFLC, and EVOA-AFLC.

	AFLC 1	AFLC 2	AFLC 3	AFLC 4	AFLC 5	AFLC 6	Mean Square
EVOA	0.014	0.011	0.093	0.114	0.0086	0.00546	0.00368
C-BO	0.023	0.003	0.112	0.126	0.0128	0.00828	0.00486
GA	0.035	0.029	0.136	0.236	0.0138	0.0136	0.01277

**Table 8:** IAE values for the algorithms MPA-PI.

	MPA
PI-1	0.064
PI-2	0.059
PI-3	0.153
PI-4	0.326
PI-5	0.0213
PI-6	0.0126
Mean square	0.02298

## Experimental setup

**Fig. 22:**
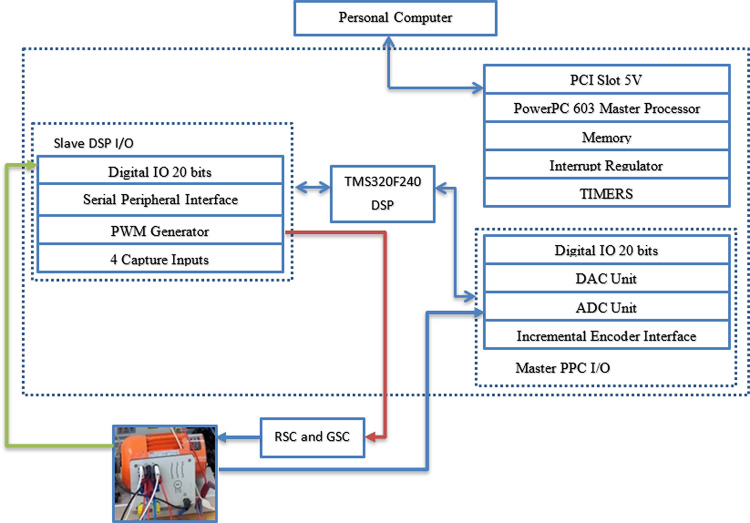
Dspace 1104 control card.

**Fig. 23:**
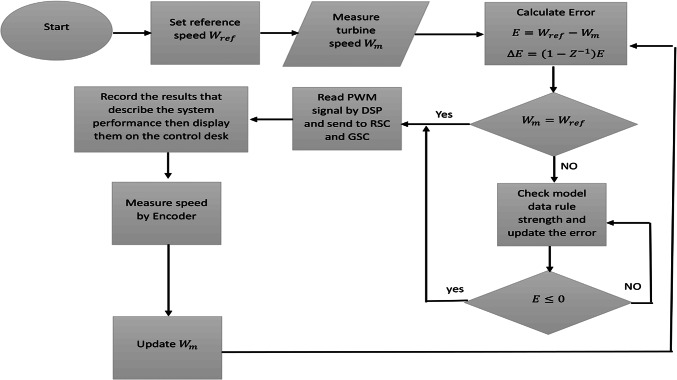
Flowchart of laboratory experiment.

Dspace 1104 card is low-cost real-time interface (RTI) which can be inserted in a personal computer. It can be integrated with Simulink software to test control systems. Input and output of the control system can be set graphically using ControlDesk 4.2. Dspace 1104 card is illustrated in Fig. 22. The experiment can be performed based on two aspects (hardware part and software part). Software part is basically DSP to MATLAB library on Simulink. The hardware part is responsibly of generating PWM signals with sampling time of 50µs. Figure 23 shows the flowchart of experimental procedures^56–59^.

**Fig. 24:**
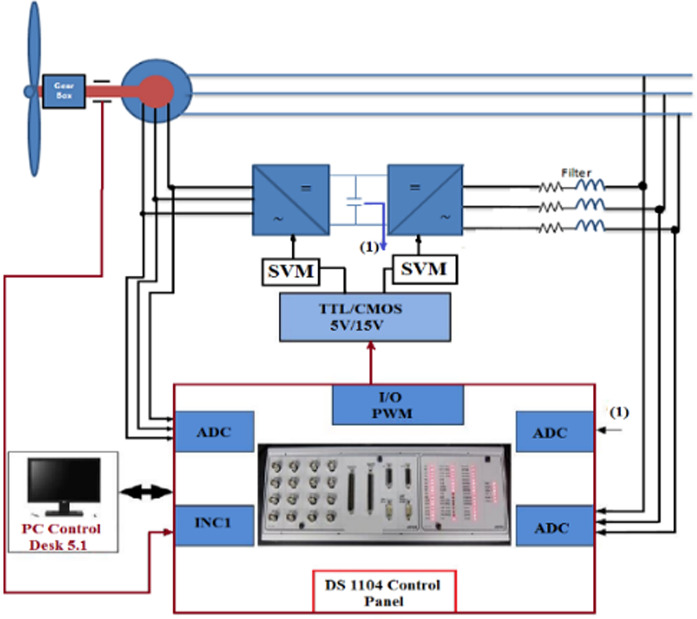
The laboratory setup of the system.

The configuration of the laboratory hardware setup is illustrated in Fig. 24. In this setup, a DC motor simulates the wind speed and serves as the prime mover, directly coupled to the DFIG. The experimental setup includes several key components:

- An incremental encoder, which functions as both a position and speed sensor. It provides 1024 pulses per revolution, supports speeds of up to 6000 rpm, and has a moment of inertia of 35 g·cm².
- A 4-pole double-fed induction machine with a power rating of 0.27 kW, voltage specifications of 230/400 V at 50 Hz, and a power factor of 1/0.75, with current ratings of 3.2/2 Amps.
- A prime mover comprising a 250 W separately excited motor, running at 3000 rpm, with a voltage rating of 180/220 VDC.

## Experimental results

**Fig. 25:**
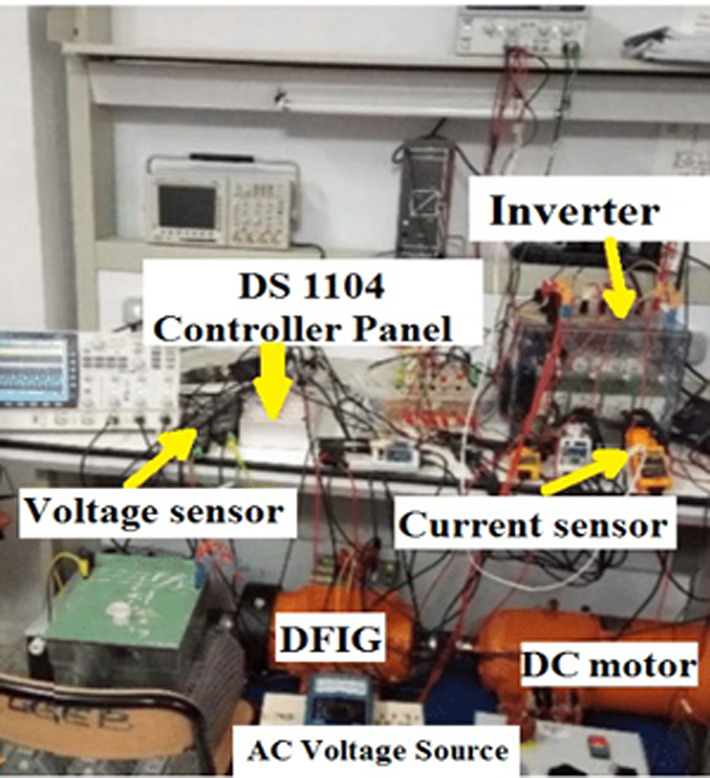
The experimental hardware wiring the system.

The experimental validation of the DFIG wind energy conversion system was carried out using the DS1104 control board, as depicted in Fig. 25. In this setup, a DC motor is connected directly to the DFIG to serve as the prime mover, simulating variations in wind speed. An incremental encoder delivers digital signals to the DS1104 control panel, enabling real-time measurement of the rotor’s actual speed.

**Fig. 26:**
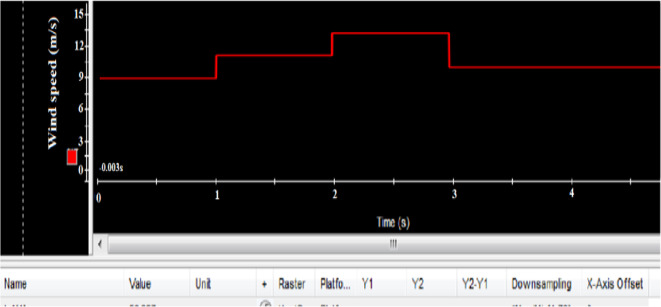
Hardware simulation wind speed profile.

The experimental setup of the DFIG system, utilizing the DS1104 control board, is shown in Fig. 25, with a comprehensive wiring diagram. The control system’s graphical interface is managed through the DS1104 RTI in Control Desk, allowing real-time monitoring of hardware simulation outcomes. As shown in Fig. 26, the wind speed was varied at intervals of t = 1s, 2s, and 3s, ranging from 9 to 13 m/s using a DC motor to simulate the wind.

**Fig. 27:**
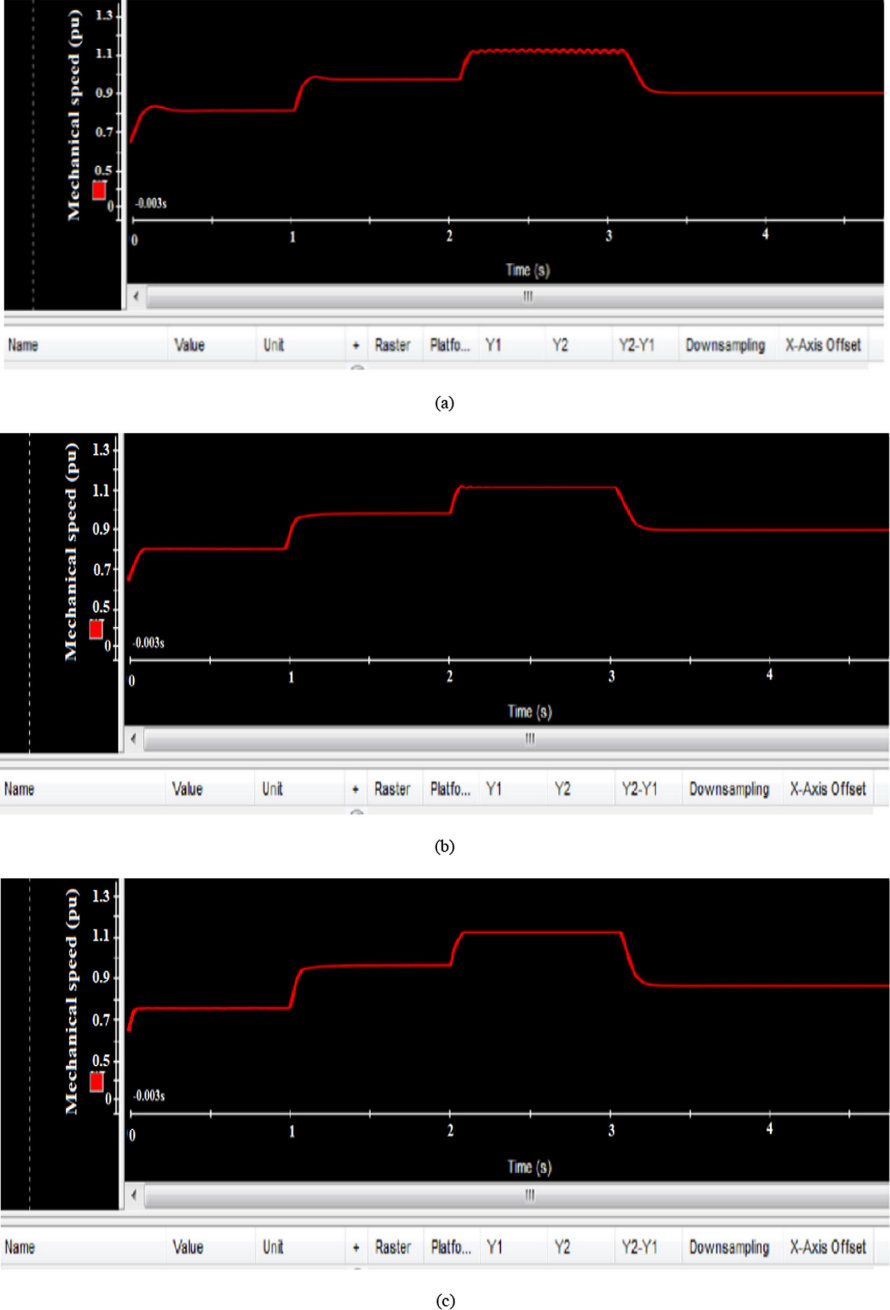
Hardware simulation mechanical rotor speed using (**a**) GA based AFLC, (**b**) C-BO based AFLC, and (**c**) EVOA based AFLC.

Figure 27 illustrates the mechanical rotor speed’s response to these wind speed variations. The EVOA-based AFLC proved to have a faster response, achieving alignment with the reference rotor speed more quickly at t = 1s, t = 2s, and t = 3s when compared to the C-BO-AFLC and GA-AFLC.

**Fig. 28:**
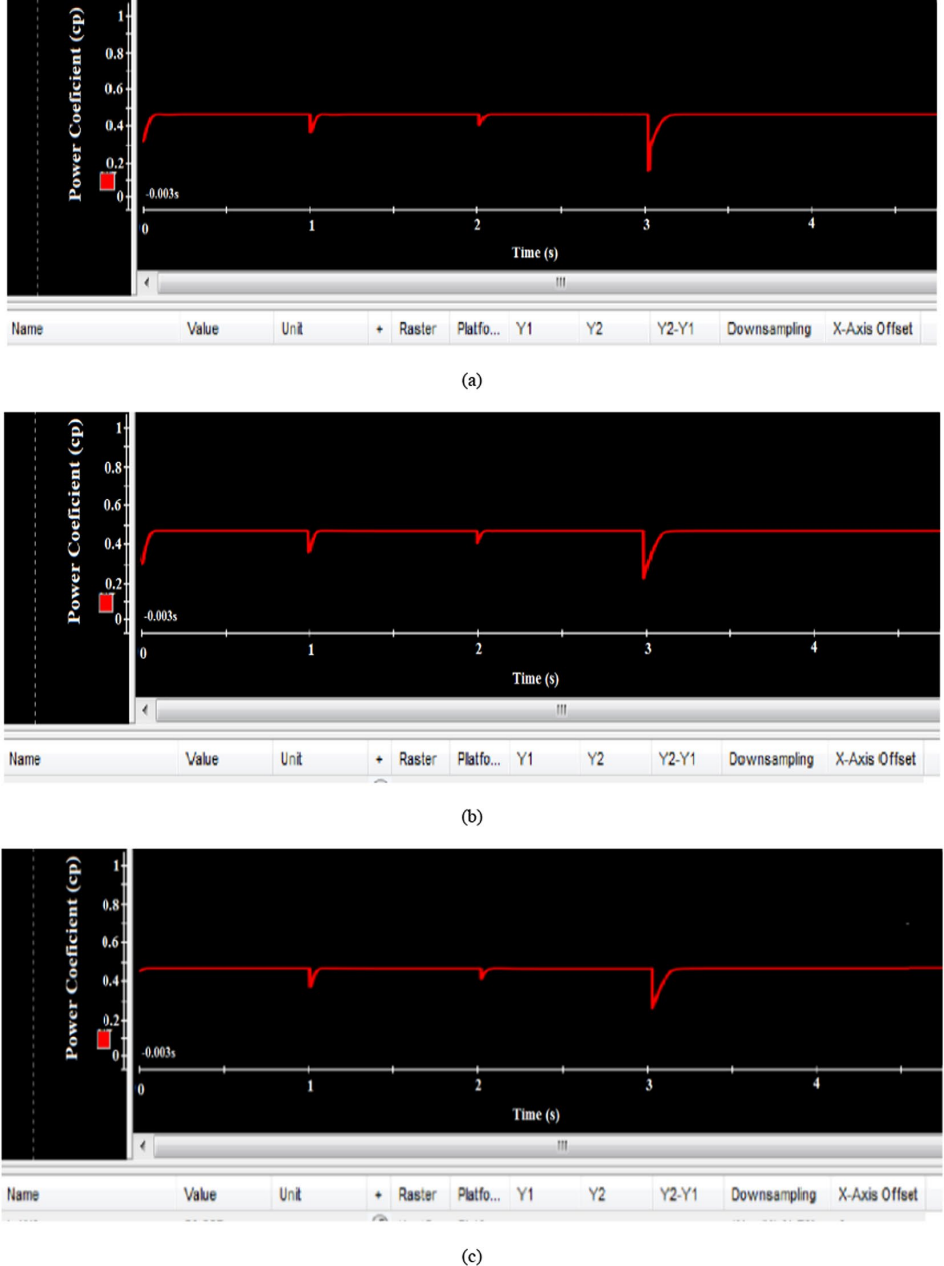
Hardware simulation power coefficient using (**a**) GA based AFLC, (**b**) C-BO based AFLC, and (**c**) EVOA based AFLC.

Figure 28(a), (b), and (c) show that the power coefficient (Cp) is maintained at its optimal value of 0.48 despite changes in wind speed. The EVOA-AFLC demonstrated a faster reaction compared to the C-BO-AFLC and GA-AFLC when responding to these fluctuations at t = 1s, t = 2s, and t = 3s.

**Fig. 29:**
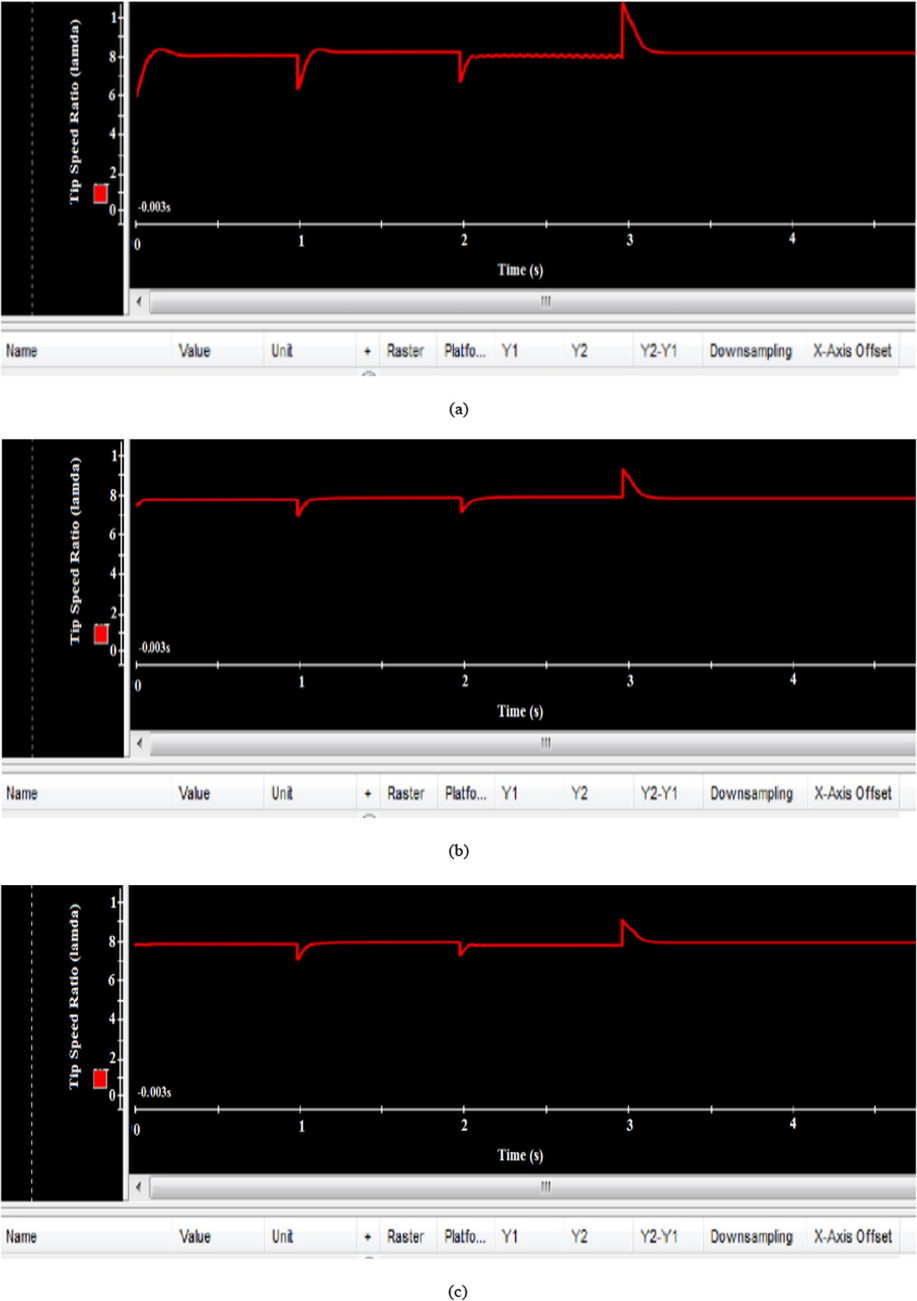
Hardware simulation tip speed ratio using (**a**) GA-based AFLC, (**b**) C-BO-based AFLC, and (**c**) EVOA-based AFLC.

Figure 29(a), 29(b), and 29(c) show that the tip speed ratio (λ) remains at its optimal level of 8.1 despite substantial wind speed changes at t = 1s, t = 2s, and t = 3s. Both the EVOA-based AFLC and C-BO-based AFLC demonstrate quicker responses compared to the GA-based AFLC, with the EVOA maintaining the desired tip speed ratio of 8.1 and exhibiting the lowest steady-state error and minimal peak overshoot at t = 3s.

**Fig. 30:**
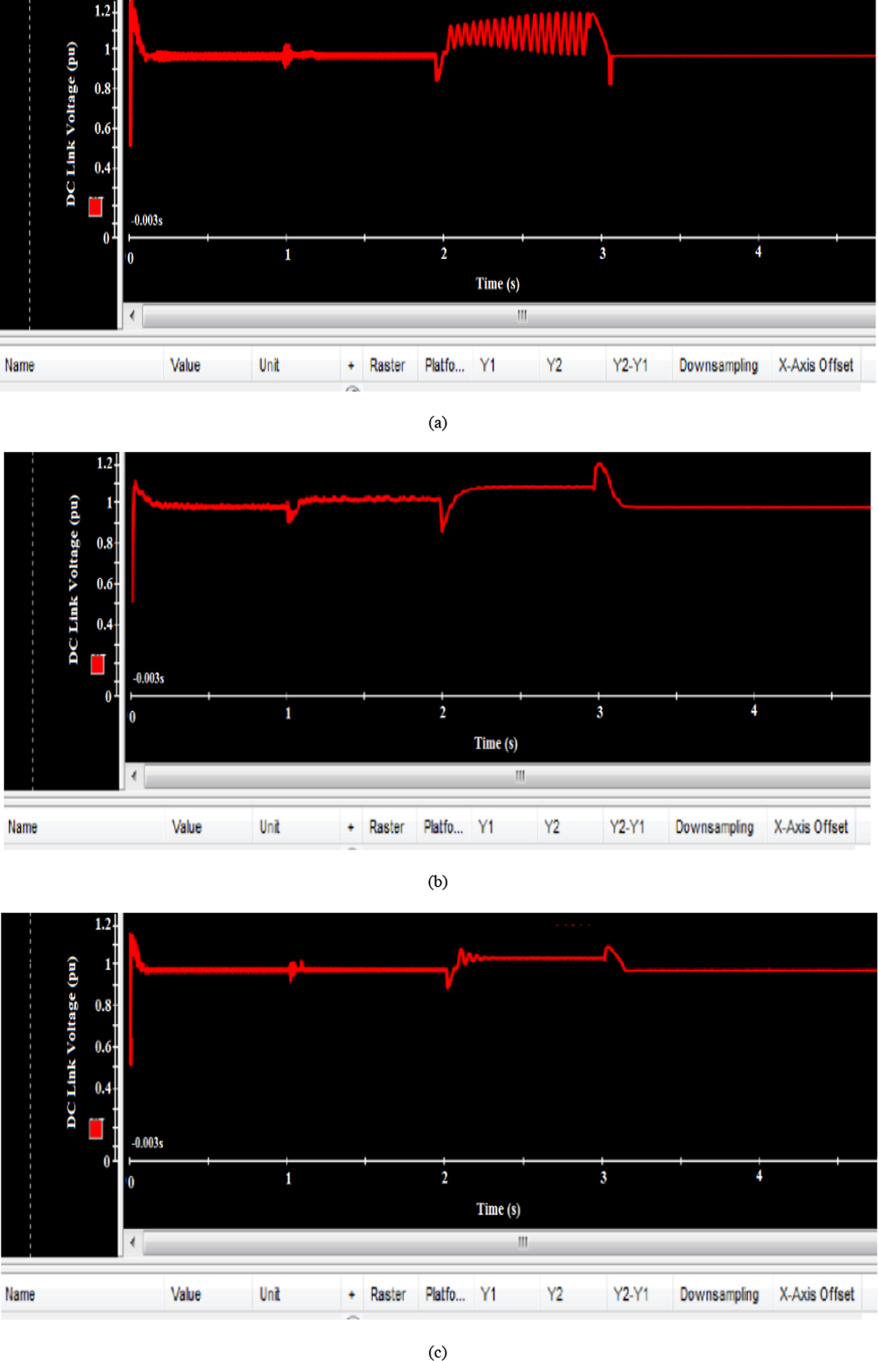
Hardware simulation tip speed ratio using (**a**) GA based AFLC, (**b**) C-BO based AFLC, and (**c**) EVOA based AFLC.

The DC link voltage responses for the various controllers are illustrated in Fig. 30. The EVOA-based AFLC exhibited a faster stabilization with fewer oscillations, and the system returned to steady-state operation more effectively following disturbances at t = 2s and t = 3s compared to the GA-AFLC and C-BO-AFLC.

The co-simulation results confirm the validity of the hardware findings by comparing them with simulation results from the Simulink/MATLAB package. The results demonstrate improved system response and tracking capabilities with the EVOA-AFLC compared to the C-BO and GA-AFLCs. The use of EVOA-based AFLC enhances tracking performance and reduces oscillations compared to the GA-AFLC. For validating results using the Dspace 1104 controller, consider the following internal and external threats to validity:

Internal Threats

1. *Instrumentation Errors*: Inaccuracies in sensors or faulty Dspace 1104 settings may introduce biases, impacting result reliability.
2. *Measurement Consistency*: Repeated tests on Dspace 1104 may vary if conditions aren’t precisely controlled, affecting result consistency.
3. *Algorithmic and Parameter Configuration*: Incorrect configuration or tuning of control algorithms may lead to incorrect results or misrepresentations.

External Threats

1. *Generalizability*: Results from Dspace 1104 may not apply to other hardware systems due to differences in controller architecture.
2. *Environmental Variability*: External factors like temperature and electromagnetic interference can influence system behavior, potentially skewing results.
3. *Real-world Application Differences*: The validation results may differ when the controller is used in field conditions versus controlled laboratory settings.

## Conclusion and future work

This article highlights the effective development of various Adaptive Fuzzy Logic Controllers (AFLCs) using the innovative Energy Valley Optimizer Algorithm (EVOA). The primary aim is to enhance the stability and performance of grid-integrated Wind Power Plants (WPPs) under varying wind conditions. Through both simulation and real-time implementation using the DSPACE DS1104 control board, the EVOA-AFLC technique was rigorously tested and validated. The experimental results align closely with the simulation outcomes, confirming the effectiveness of the model under identical runtime and wind-speed profiles. Optimal gain factors for six AFLCs were determined by minimizing the integral square error, which was used as the fitness function for optimizing the EVOA and C-BO algorithms. The EVOA-AFLC outperforms other approaches significantly, achieving a 71.2% reduction in mean square error compared to the GA-AFLC, 24.4% compared to the C-BO-AFLC, and an impressive 84% compared to the MPA-PI. These results underscore the superior performance of the EVOA compared to other algorithms for the same WPP setup. The EVOA-AFLC demonstrates notably reduced oscillations, faster transient responses, and improved efficiency over the C-BO-AFLC and GA-AFLC. Specifically, it enhances speed tracking by 86.3% compared to the MPA-PI, 56.36% compared to the GA-AFLC, and 39.3% compared to the C-BO.

In future work, we plan to investigate the proposed EVOA-AFLC control model on oscillating water column power plant. Similar to any other controller that uses integration, the AFLC could encounter difficulties with wind-up in cases involving wind that have irregular profiles. Finally, the robustness of the proposed controller in terms of introducing noise into the wind speed signal will be investigated in future work.

## Data Availability

The datasets used and/or analysed during the current study available from the corresponding author on reasonable request.

## Abbreviations

R: radius of the blade
ρ: the density of air
v_w_: the speed of wind
C_P_ (β,λ): power coefficient
λ: the tip speed ratio or TSR
β: the pitch angle of the blades
n: the entire number of solution candidates in the search space
d: the considered problem dimension
$y_{{\text{i}}}^{{\text{j}}}$: the jth decision variable to determine the starting location of the ith candidate
$y_{{\text{(i,min)}}}^{{\text{j}}}$ and $y_{{\text{(i,max)}}}^{{\text{j}}}$: represent the minimum and maximum bounds of the jth variable in the ith particle
rand: random number between 0 and 1
NEL_i_: the neutron enrichment level of the ith particle
EB: the enrichment bound of the particles in the universe
*SL*_*i*_: the stability degree of the ith particle
$Y_i^{New1}, Y_i^{New2}$: are the newly generated particles in the universe
*Y*_*i*_: the ith solution candidates’ current location vector in the search space
*Y*_*BS*_: the vector location of the candidate with the optimum degree of stability
$D_i^k$: the distance between the ith candidate and the kth nearby candidate
